# Biomedical Science to Tackle the COVID-19 Pandemic: Current Status and Future Perspectives

**DOI:** 10.3390/molecules25204620

**Published:** 2020-10-11

**Authors:** Camilo Zamora-Ledezma, David F. Clavijo C., Ernesto Medina, Federico Sinche, Nelson Santiago Vispo, Si Amar Dahoumane, Frank Alexis

**Affiliations:** 1 School of Physical Sciences and Nanotechnology, Yachay Tech University, Urcuquí 100650, Ecuador; emedina@yachaytech.edu.ec; 2 School of Biological Sciences and Engineering, Yachay Tech University, Urcuquí 100650, Ecuador; david.clavijo@yachaytech.edu.ec (D.F.C.C.); fsinche@yachaytech.edu.ec (F.S.); nvispo@yachaytech.edu.ec (N.S.V.); falexis@yachaytech.edu.ec (F.A.)

**Keywords:** diagnostics, prevention, nanomedicine, vaccines, treatment, clinical trials

## Abstract

The coronavirus infectious disease (COVID-19) pandemic emerged at the end of 2019, and was caused by the Severe Acute Respiratory Syndrome Coronavirus 2 (SARS-CoV-2), which has resulted in an unprecedented health and economic crisis worldwide. One key aspect, compared to other recent pandemics, is the level of urgency, which has started a race for finding adequate answers. Solutions for efficient prevention approaches, rapid, reliable, and high throughput diagnostics, monitoring, and safe therapies are needed. Research across the world has been directed to fight against COVID-19. Biomedical science has been presented as a possible area for combating the SARS-CoV-2 virus due to the unique challenges raised by the pandemic, as reported by epidemiologists, immunologists, and medical doctors, including COVID-19’s survival, symptoms, protein surface composition, and infection mechanisms. While the current knowledge about the SARS-CoV-2 virus is still limited, various (old and new) biomedical approaches have been developed and tested. Here, we review the current status and future perspectives of biomedical science in the context of COVID-19, including nanotechnology, prevention through vaccine engineering, diagnostic, monitoring, and therapy. This review is aimed at discussing the current impact of biomedical science in healthcare for the management of COVID-19, as well as some challenges to be addressed.

## 1. Introduction

The coronavirus infectious disease (COVID-19) pandemic emerged at the end of 2019, and was caused by the Severe Acute Respiratory Syndrome Coronavirus 2 (SARS-CoV-2), a new virus genetically related to the coronavirus responsible for the Severe Acute Respiratory Syndrome (SARS) outbreak of 2003 [1,2,3,4,5]. On 1 January 2020, the World Health Organization (WHO) activated its incident management support team after the notification of the first cases, followed by an official publication on 5 January 2020. On 22 January 2020, the first COVID-19 case outside China was reported. The WHO gave their first public remark about the emergence of the COVID-19 disease on 4 February 2020, to present some preliminary results, with 20,471 cases in China and 425 deaths (99% of all cases reported), and to warn the world about the onset of a global pandemic. At that stage, there were only 176 cases reported outside China [6]. On 10 February 2020, more information about the mortality was released, with a rate of 2%, suggesting that, if the virus can infect rapidly, it could have a severe effect on the number of deaths. On 11 February, the WHO presented a series of questions that needed to be addressed to respond to the pandemic. On 17 February, more data was made available on population risk factors, such as age and pre-existing conditions. On 14 February, more information was reported about the non-significant change of the genetic makeup of the virus and the recovery time of patients. However, there was a sudden increase of cases in the world, including in Italy, Iran, and the Republic of Korea. For the first time, there were serious concerns of a pandemic in progress. On 27 February, there were cases reported in 44 countries, in all continents, and the WHO recommended that all countries make efforts to detect the virus early. More than 20 vaccines, and other therapeutic approaches, started to be tested in clinical trials. On 3 March 2020, it was reported that 1% of the cases did not have symptoms, suggesting that the infection could be more challenging to contain without rapid diagnostic methods. At this stage, the death rate comparison between seasonal flu (influenza) and COVID-19 was a factor of three, with a 3.4% death rate for COVID-19. On 4 March, the first signs of community transmission appeared in some countries. A month later, in the first public report from the WHO about COVID-19, the number of reported cases reached 100,000, exhibiting exponential growth [6]; thus, research priorities were listed, including the natural history of the virus, epidemiology, vaccines, diagnostics, treatments, clinical management, ethical considerations, and social sciences. The rapid progression of the virus suggested that medical devices and therapeutics would be essential to manage the number of patients arriving at hospitals. By 13 March, more than 100 countries reported cases of COVID-19, with an increase by a factor of thirteen outside of China, and almost 5000 deaths worldwide, which concluded that COVID-19 could be considered a pandemic. The WHO stressed the need to detect, prevent, and treat COVID-19 through innovation. At that time, it was reported that the pandemic center shifted from China to Europe, and more than 80% of the total active cases were reported from Western Pacific and Europe due to the escalating epidemic. The first vaccine (mRNA1273) clinical trial in the USA started less than 60 days after the genetic sequence of the virus had been made public on 27 February 2020. By 25 March, the WHO reported an increase of funding for diagnostic and therapeutic solutions. On 26 March, the results of the pandemic expansion showed that it took 67 days for the first 100,000 COVID-19 infected cases to be reported compared to 11 days for the second 100,000 cases, 4 days for the third 100,000 cases, and only 2 days for the fourth 100,000 cases, highlighting the exponential growth of the pandemic [6]. On 3 April 2020, more than 1 million cases and 50,000 deaths were reported worldwide. The fatality rate is estimated to be 10 times higher than that of influenza. By 16 April, the number of cases and deaths doubled within only 2 weeks, and 40,000 deaths were numbered within a single week. Four months later, more than 18 million cases of COVID-19 had been reported, including more than half a million deaths, and it continued to climb higher. The pandemic has not slowed down, and more than 160,000 cases have been reported each day [6].

**Figure 1:**
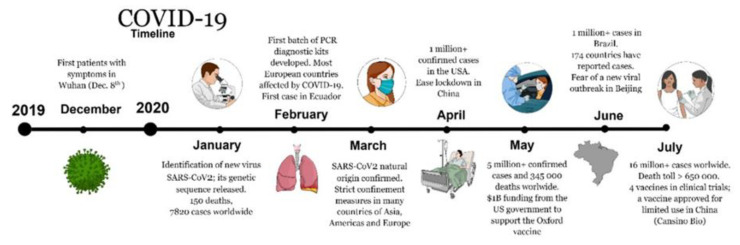
Coronavirus infectious disease (COVID-19) timeline highlighting the most striking events from December 2019 to July 2020.

Pandemics have played an important role in society adjusting to a new reality, by developing new solutions [1,2,5,7]. According to the United States Centers for Disease Control and Prevention (US CDC) data, since the beginning of the human immunodeficiency virus (HIV) pandemic in 1981, more than 30 million people have died, and approximately 40 million individuals live with acquired immune deficiency syndrome (AIDS). The HIV virus can evade the immune system and remain in the body for life. New HIV infections have been reduced by 40% since the peak in 1997. Moreover, the number of deaths has been reduced by more than 50% since the peak in 2004. In 2002, severe acute respiratory syndrome (SARS) caused by a virus known as SARS-CoV, infected an estimated 8000 people, with a mortality rate of 10%. More recently, in 2009, the swine flu, caused by swine influenza virus (SIV), affected more than 60 million people, with a transmission rate of about 1.4–1.6 people, according to the US CDC. The total number of deaths reached approximately 12,000, with a mortality rate of about 0.02%. In 2012, the Middle East Respiratory Syndrome (MERS), caused by the MERS-CoV virus, infected 3000 people, and resulted in less than 1000 deaths. The mortality rate in the population infected by these viruses varies significantly due to their different mechanisms of infection, expansion rate, absence of natural immunity, surveillance mechanisms, early diagnostics, prevention methods, and access to efficient therapy. COVID-19 differs from the abovementioned viruses in terms of infectious period, transmissibility, clinical severity, and extent of community spread. Compared to influenza, the mortality rate is at least 30 times higher, and its reproductive number (2–2.5) is higher than that of the swine flu. According to Prof. Michael Farzan, the SCRIPPS Research Institute virologist who discovered the angiotensin-converting enzyme 2 (ACE2) receptor binding site of SARS-CoV-2 [8], the level of complexity is significantly lower due to its low resistance to our immune system, suggesting that a single vaccine would work to prevent COVID-19 [9]. However, considering the reported mutations specific to geographical regions, it may suggest that one vaccine may not be effective against all strains. The challenge remains in containing its high transmission efficiency until natural immunity is developed and treatments are made available. Therefore, the magnitude of the mortality rate has created an emergency crisis to rapidly provide solutions. Figure 1 presents a timeline of the most important events in the current COVID-19 pandemic, from December 2019 to July 2020.

Biomedical science and engineering have been presented as possible areas to serve medical science to combat SARS-CoV-2, due to the unique challenges reported by epidemiologists, immunologists, and medical doctors, including survival, symptoms, protein surface composition, and infection mechanisms [1,3,4,10]. These multidisciplinary engineering concepts are applied to design and develop prevention methods, diagnostics, monitoring, and therapies. Here, we review the different scientific and technological aspects from various approaches, such as nanotechnology, vaccine engineering, pharmaceuticals, medical devices, computation, bioimaging, sensing, etc., deployed by the scientific community, to tackle the propagation of SARS-CoV-2, detect and monitor the evolution of this disease, and, ultimately, cure it.

## 2. Open Access to Designs and Patents

In the first SARS case (2002), 773 patent families were registered, and it was 10 times less for MERS (2012). However, despite a concerted global effort, no effective treatment or vaccine for SARS was found [11,12]. The current emergency situation has changed the scope of open access and exchange of designs and patents due to the unprecedented urgent needs. Scalability and mass production are key factors driving these new collaboration mechanisms [13]. In the current context of COVID-19, researchers are working against time. According to a recent report, more than 160 medications at different stages of development are being investigated to fight COVID-19 [14]. While countries have closed their frontiers, science and research expand theirs, promoting worldwide multidisciplinary, collaborative work. As far as we know, this unprecedented disease opens a wide range of opportunities to gather scientists and experts around the entire world as never seen before, working simultaneously towards a common goal [15].

On 10 January 2020, scientists from China uploaded the first genetic sequence of SARS-CoV-2 that was shared with the WHO two days later. The early availability of the genetic sequence allowed laboratories around the world to start developing diagnostic test kits and launch research on antiviral drugs and vaccines [16]. Since then, thousands of SARS-CoV-2 sequences from around the world have been uploaded to online databases, such as GenBank and the Global Initiative on Sharing All Influenza Data (GISAID). These genetic sequences have helped to track the spread of SARS-CoV-2, determine successful containment strategies, and monitor the occurrence of viral adaptive mutations [16]. In spite of several national and international research groups that are currently working intensively on their development to prevent and treat COVID-19, vaccines are not yet available. At the present time, an urgent challenge also deals with the development of effective prevention and treatment strategies for the outbreak. One of the approaches used so far consists of using existing drugs. This option has been moderately accepted as it allows to save time and money with a satisfactory proved efficiency, if compared to the development of new drugs or vaccines [17]. For its part, the U.S. Food and Drug Administration (USFDA or FDA) has authorized the use of in vitro diagnostic products, and not yet approved test kits based on SARS-CoV-2 antibodies to face the current COVID-19 emergency. In addition, the use of hydroxychloroquine sulfate and chloroquine phosphate together with Remdesivir (sold under the name Veklury) was also approved to treat patients with severe COVID-19 symptoms, when a clinical trial is unavailable or not feasible [18]. Excelra made public its drug database for COVID-19, which includes therapeutic antibodies, cytokines, and nucleic acid-based therapies targeting virus gene expression, as well as various types of vaccines [19]. Pharmaceutical companies, such as AbbVie, have waived their patent rights on the use of Lopinavir© and Ritonavir© antivirals [20]. Pfizer and BioNTech have teamed up to develop a vaccine against COVID-19 and respond to the global demand to possibly supply millions of vaccine doses by the end of 2020 [21].

During a severe crisis, such as the present one, efficient and adequate access to intellectual property rights seems to be essential to stop the global spread. The government of Costa Rica created a voluntary group to democratize coronavirus-related intellectual property (IP) rights, copyrights, designs, licenses, and patents based on the example of the Medicines Patent Fund (MPP) [22]. In this context, patents can be a valuable tool and source of knowledge for the current and future coronavirus outbreaks [13]. In Canada, companies are not only sharing intellectual property, but also allowing others to replicate their products, including software tools, basic supplies, and protective and medical equipment [23]. In the area of medical devices, Medtronic has shared the design of its Puritan Bennett (PB) 560 portable ventilator to enable qualified manufacturers to produce it [24]. On the other hand, the Italian company Isinnova created a three-dimensional (3D)-printed Venturi valve, without the original drawings, by reverse engineering, due to the lack of supply of this key component for oxygen masks [25]. Hewlett Packard Enterprise (HPE) together with Amazon, IBM, Facebook, Intel, and Microsoft have also made a commitment to make their intellectual property available to diagnose, prevent, contain, and treat the coronavirus through the Open COVID Pledge initiative to open proprietary technologies for the public. Patents encompass developments in diverse areas, such as biomedical devices, artificial intelligence, mathematical models, algorithms, computer hardware, blockchain technology, cloud computing, quantum computing, and security [26,27,28]. Large companies have also joined the cause of the COVID-19 combat, such as Bauer, Dyson, General Motors, Ford, General Electrics, UAW, and 3M, by changing their regular production for protective masks and ventilators [29]. Roche and Gilead initially attempted to maximize their market position by strengthening the property rights, sparking a negative response from the global community, provoking the flexibility of company’s positions vis-à-vis their IP resources [30].

**Figure 2:**
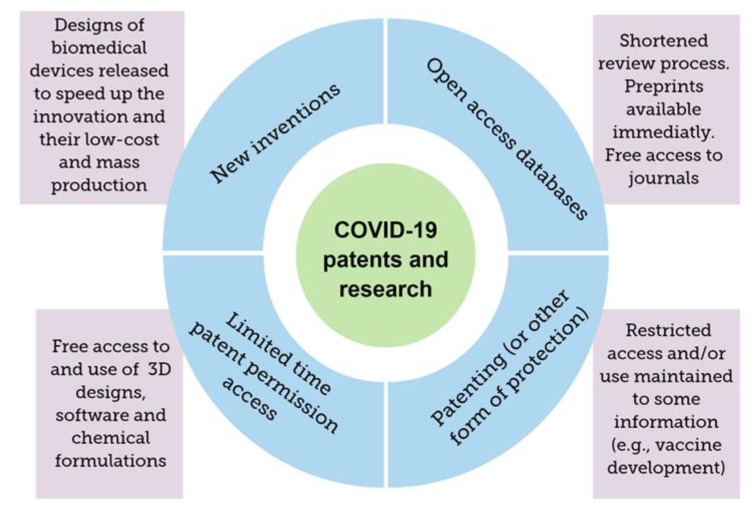
Dissemination and use of research about COVID-19: research and development and access to patents.

An open call from governments worldwide supported the decision of many scientific journals and publishers of an open access model that allows the use of free archives of research in medical sciences, clinical trials, and preliminary research. As an example, medRxiv, chemRxiv, and bioRxiv, are some of the most common online archives that share academic research before they are peer-reviewed and published in journals [15]. Overall, the exchange of patented technology and access to databases and open resources mark a new paradigm, and have been a key factor in accelerating both research and development processes, especially due to the global impact of the pandemic. Figure 2 summarizes the most outstanding open access initiatives.

## 3. Biomedical Devices

Researchers and companies worldwide have changed their focus or production lines to develop products that meet the needs of medical personnel and patients motivated by the COVID-19 urgency and satisfy the demand of medical supplies and emergency care equipment, with special emphasis on personal protective equipment. Since the beginning of the pandemic, the distribution lines were affected. China, being the largest producer of personal protective equipment (PPE), was the first country to close its borders, making it impossible for other countries to get supplied with large amounts of protective equipment [31]. As the global imports were reactivated by the emergency, the high demand caused the stocks and supplies to run out and this led to protection being unavailable. According to the WHO, it is estimated that, monthly, 90 million protective masks, 76 million gloves, 30 million biosafety suits, and 1.6 million glasses or face shields are needed [31].

In addition, the WHO guidelines are very strict regarding the mask specifications that must fulfill the minimum requirement of filtering 99.9% of microorganisms and viruses [32]. Surgical masks do not meet these requirements since they only filter 67% of pathogens due to the pore size; therefore, their usefulness is reduced to less than an hour [32]. Innovations, such as isolation hoods that cover half of the body of patients infected by COVID-19, developed by the Harvard University School of Design, protect not only the patient, but also front-line medical personnel [33]. The demand for respirators and oxygen tanks is 10 times greater than the capacity of health systems, on average. Moreover, their prices have also increased due to the sustained demand and competing governments to buy these devices. Vanderbilt researchers (USA) are investigating the usefulness of implementing a remote monitoring system within the health centers that house patients with COVID-19 to avoid unnecessary contact [34]. They labeled this tool as electronic Personal Protective Equipment (ePPE), which differs from telemedicine, in which the doctor is available to immediately attend patients in need.

Hundreds of universities and nonprofit organizations have come together to produce large-scale personal protective equipment using 3D printers from face shields to masks and biosafety suits [31]. This protective equipment has different characteristics including the potential to remove viruses from the surface through antiviral coatings. This technology has been also used to fabricate hand-free door openers, disinfection equipment, emergency fans, air filters, robots that disinfect high-risk areas, wireless sensor to detect COVID-19 early symptoms, smart watches to measure blood oxygen saturation, valves for respirators, and spare parts for medical equipment that have been damaged due to lack of use. In addition, the FDA has approved the emergency use of devices that were not created with the objective of treating patients with COVID-19, but that can be very useful given the circumstances [18]. Their use can be decisive in the evolution of the disease in patients with severe symptoms. Among the granted permissions are blood purification devices, hemodialysis devices, decontamination systems for personal protective equipment, infusion pumps, remote or wearable patient monitoring devices, Respiratory Assist Devices (RAD), ventilators, and ventilator accessories. In this context, the Hemolung Respiratory Assist System (RAS) is a very promising device. It is used for extracorporeal carbon dioxide removal and treatment of patients with pulmonary failure due to serious infections, transplants, or other pathologies [35]. This technology has proven to be very efficient to restore the oxygen saturation levels in critically ill patients with COVID-19.

**Figure 3:**
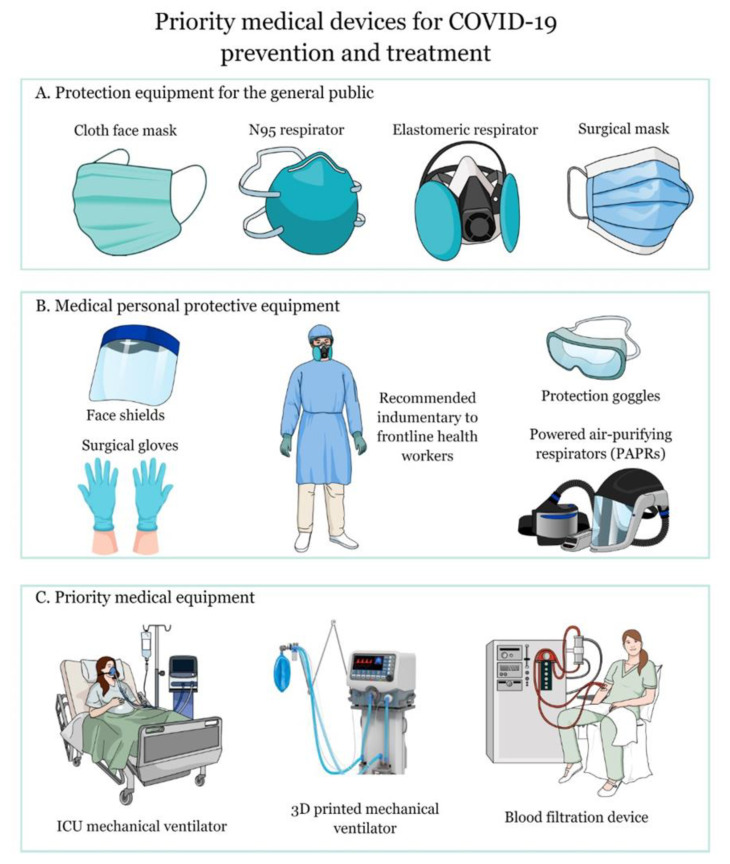
Priority medical devices for COVID-19 prevention and treatment. (**A**) Face masks are the most used protection equipment in the general population, but for frontline health workers, special filter masks should be used to prevent infection. (**B**) Face shields, gloves, filter respirators, plastic boots, protection goggles, and hospital gowns are recommended for medical staff members. (**C**) Mechanical ventilators are priority devices for the intensive care unit, but other devices, such as blood filtration devices, have been approved by the U.S. Food and Drug Administration (FDA) for emergency use, to avoid complications due to an excess of cytokines in patients with an overloaded immune response. Most of these devices have been replicated by professionals and volunteers, with 3D models made available for the general public to meet the demand.

The development of software and technological applications, such as telemedicine to track the evolution of the virus in the population, has gained attention due to the risk of infection and rapid spread. On the other hand, the restructuring of public spaces is a reality, and companies are beginning to adapt to the new normality that demands distancing measures to get out of quarantine [36]. Locating infected people being a key aspect to stop the progress of the virus—authorities have taken preventive measures, isolating the relatives of a person diagnosed positive with COVID-19, in addition to contacting people who were close to the affected individual in the days prior to diagnosis [37]. For this, digital tools have been developed facilitating the work of governments. Digital monitoring or contact tracking applications have different approaches; sometimes, they use the integrated Global Positioning System (GPS) of the phones to geographically locate people infected with the virus and alert the residents of the sector; other developers use the phone’s Bluetooth to connect it with the closest devices, to alert the presence of someone with COVID-19 symptoms or at risk of infection [2]. Bracelets with quick response (QR) codes have also been implemented to prevent people from leaving the epidemiological fences. In addition, applications have been developed with specific questions so that users can know if they have any symptoms of COVID-19 and learn what to do if they get infected [37]. An example is the application known as Healthy Together, developed by the Utah Government. Moreover, Trace Together and COVID Safe are examples of government contact tracing applications from Singapore and Australia, respectively [38]. Table 1 and Figure 3 summarize the most outstanding essentials in the field of biomedical devices to tackle the current COVID-19 pandemic, as well as future pandemics.

## 4. Prevention of COVID-19 Infection Using Biomedical Science

There is no effective vaccine for either MERS or SARS and, therefore, stringent public health measures were necessary to contain these outbreaks [50,51]. Although social distancing measures might be effective in containing COVID-19, they have not been enforced fast or efficiently enough to contain and stop its global spread [52,53]. Temperature and humidity have been reported to play important roles in the activity of COVID-19, particularly at temperatures of 20–25 °C with a humidity of at least 40% [54,55,56]. To this end, the CDC has issued safety guidelines for droplet barrier precautions, environmental hygiene, and overall infection prevention practices to ensure minimal risk of COVID-19 infection [57]. Moreover, the success of prevention or treatment of many respiratory infections including COVID-19 depends on the efficacy of the decontamination substances or protection equipment being used [58,59,60]. This is certainly true for the COVID-19 outbreak, where research is being carried out at the same time the pandemic is unfolding. However, early countermeasures of protection and diagnosis significantly slow the spread of COVID-19 to a level that is manageable within the capacity of the healthcare system.

Recent work has reported the efficacy of household or alcohol-based disinfectants for the decontamination of hospital material in places with COVID-19 patients [61]. However, some of the limitations of this class of products include unregulated dosages, overuse, and its potential hazard for the environment and health [62,63,64]. In this context, engineered nanomaterials represent suitable candidates to significantly contribute to the prevention of both environmental contamination and contagion by SARS-CoV-2. The attributes of these materials include antiviral and virucidal properties, as well as their effectiveness in small doses with a minimal toxicity [65,66,67,68,69].

### 4.1. Nosocomial Infections in Healthcare Facilities

Healthcare facilities represent high-risk sites for infection with SARS-CoV-2; therefore, healthcare workers must consider themselves at an elevated risk of infection [60,70,71]. A nosocomial infection, also called Hospital-Acquired Infection (HAI), is an infection that can be acquired during hospitalization and includes infections transmitted from different sources within the hospital (e.g., contaminated surfaces) [72]. Nosocomial outbreaks have recently been reported in different healthcare units in hospitals for respiratory syndromes, including SARS [59,60,73]. As of today, in countries with the highest confirmed cases of SARS-CoV-2, such as the USA, Brazil, Russia, China, Italy, and India, more than 90% of healthcare workers have been infected, and 0.3–0.6% have died [74,75]. These reports underscore the need for prevention of cross-infection among healthcare providers and patients with COVID-19. Since SARS-CoV-2 spreads by droplets and contact, the CDC recommends the use of personal protective equipment (PPE), including a gown, gloves, masks (N95), and eye wear in the care of all patients with respiratory symptoms, to protect the health of caregivers [76].

Respiratory droplets from viruses can be classified into large droplets (>5 µm in diameter), which can precipitate rapidly to the ground, and small droplets (≤5 µm in diameter), which can evaporate into an aerosol form called “droplet nuclei” [77,78,79]. This aerosol form is thought to remain suspended in the air; thus, increasing the chance to be inhaled [75]. This represents the first route of transmission of the coronavirus [77,78,79,80]. To prevent respiratory transmission due to SARS-CoV-2-containing aerosols, it has been advised to maintain, at least, a 3-foot (1 m) to 6-foot (2 m) separation from a person with symptoms of COVID-19 [81,82,83]. However, studies modeling human sneezes using turbulent gas clouds have indicated that pathogen droplets of all sizes can travel 23 to 27 feet (7–8 m) [84,85]. Moreover, in a study by van Doremalen et al., it was reported that SARS-CoV-2 droplets can remain viable in aerosol for up to 3 h [86]. The second route of transmission is through surfaces contaminated by the large droplets [87,88], which can be transported by hands onto mucosal membranes (e.g., nose, mouth, and eyes) [60,70]. This route of transmission is related to studies reporting the stability of SARS-CoV-2 on a number of different surfaces and aerosols in healthcare facilities [86]. Whereas the first route of transmission has also been observed in common respiratory viruses (influenza and parainfluenza viruses, respiratory syncytial virus, adenoviruses, and coronaviruses) [89,90,91,92], the coronaviruses are equipped with a lipid-containing envelop that may play a key-role in their survival under different environmental conditions, and on various surfaces [93,94]. In this regard, studies have reported that enveloped viruses can remain infectious and survive under different environmental conditions for days to weeks at room air temperatures (4–25 °C) and varying relative humidity (20–50%) [54,55,56]. Furthermore, the stability of the enveloped viruses at low air temperatures has been associated with infectious seasonal outbreaks during the winter season, characterized by cold and dry conditions [95,96].

By now, it has been documented that the human coronavirus can remain infectious at least 5 days on common non-biocidal surface materials, such as Teflon (PTEE), polyvinyl chloride (PVC), ceramic tiles, glass, silicone, and stainless steel [97]. Likewise, in a recent study, the viability of SARS-CoV-2 titers on different surfaces and in the air was investigated using a Tissue Culture Infectious Dose at 50% (TCID50) assay [80]. As a result, this work showed that SARS-CoV-2 had its longest stability on plastic surfaces with a reduction of 103.7 to 100.6 TCID50 per mL of medium after 72 h, while a similar TCID50 reduction was found for stainless steel after 48 h [80]. The least stability of SARS-CoV-2 was observed on copper on which no viable viruses were detected after 4 h, followed by cardboard on which no viable viruses were observed after 24 h [80]. Likewise, the coronavirus SARS-CoV (2002–2003 outbreak) retained its infectivity for up to 9 days compared to the previously identified human coronavirus (HCoV) 229E that lost its infectivity within 24 h on polystyrene surface [98]. The HCoVs 229E and OC43 have also been reported to remain infectious on various surfaces (aluminum, sterile latex surgical gloves, and sterile sponges) for 1–3 h [99]. Hence, the strategies to eliminate the biocontamination of PPE and devices by SARS-CoV-2 in healthcare facilities should focus on reducing the adhesion and transport of respiratory droplets from infected patients to other persons present on-site [87].

### 4.2. Protective Personal Masks

The reduction of the coronavirus persistence on PPE surfaces can be accomplished by preventing the adhesion of the respiratory droplets onto the material surfaces [100,101]. To this end, metal oxides have been employed on different types of surfaces to induce biocidal effects over pathogens, as it is discussed below [65,66,67,69,102,103,104,105,106,107,108,109,110,111,112,113]. Copper oxide (CuO) is one of the compounds that has been used to design PPE with biocidal properties, such as respiratory face masks [67,68,69,114]. This type of equipment [115] is the first line of defense to reduce the spread of respiratory viruses according to the WHO and CDC [51,116,117,118]. However, as more infectious viruses, such as SARS-CoV-2, can spread through droplets deposited on surfaces from healthcare facilities and public spaces, it is urgent to develop improved designs of existing protective equipment, such as masks and other types of cloths, to provide safety to healthcare workers and the public.

**Figure 4:**
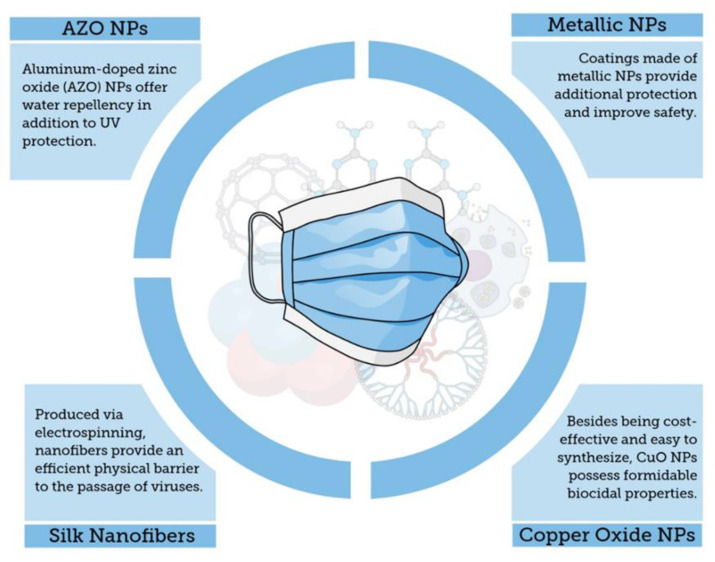
Summary of principal mask coatings based on nanoparticles to prevent pathogen adhesion to surgical masks.

Specifically, in a study about the efficacy of the impregnation of CuO particles into disposable US National Institute for Occupational Safety and Health (NIOSH) N95 respirator masks to deactivate human influenza A virus (H1N1) and avian influenza virus (H9N2), the titers were assessed and TCID50 values were reported [69]. The CuO particles were impregnated into the exterior and interior layers of the mask at 2.2% and 2.0% of *w*/*w*, respectively [69]. After comparing the recovered H1N1 and H9N2 viruses from both copper-free masks and copper-impregnated masks, it was found that the TCID50 values for both viral titers were >5-fold lower in masks with CuO than the copper-free masks. The mechanism of virus deactivation is related to the interaction between the copper ions from the impregnated mask layers and the virus titers entrapped in the mask. Moreover, the CuO impregnation did not alter the filtration capacities of the masks, and the amount of copper eluted to the air from the masks were >105-fold lower than the respiratory copper permissible exposure limit recommended by the U.S. Occupational Safety and Health Administration (OSHA) [69]. Likewise, copper iodide nanoparticles (CuI NPs) have also been investigated for applications in filters, face masks, and protective clothing [67]. To assess this potential, Fujimori et al. tested the virucidal property of CuI NPs at different aqueous concentrations (*w*/*v*) against H1N1 using a model mammalian cell line [67]. The authors reported that the virus titer was decreased due to the presence of CuI NPs in a dose-dependent manner with a half maximal effective concentration (EC50) of 17 µg mL^−1^ (0.0017%, *w*/*v*) after 1 h treatment. Furthermore, the authors documented the mode of action of CuI NPs against H1N1 which involved the conversion of CuI into Cu^+^ under aqueous conditions; the Cu^+^ triggered the generation of ^•^OH that exerted an antiviral activity against the influenza virus by degrading functional proteins, such as hemagglutinin and neuraminidase [67]. Similar modes of action by copper against other classes of virus, such as noroviruses, have been reported in which the generation of reactive oxygen species (ROS) have also been associated with the virus inactivation due to the copper toxicity on the viral encoded proteins [112]. The generation of ROS, including ^•^OH, has also been reported as the main mechanism of the antiviral activity in other copper compounds (e.g., Cu_2_O) [113]. In a similar approach, Zhong et al. reported that reusable and recyclable graphene-impregnated masks could be readily fabricated. These PPE showed enhanced superhydrophobic performances providing better protection toward incoming respiration droplets which can be the virus vector. These devices have been fabricated by functionalizing commercially available masks with graphene nanosheets and exhibited unprecedented self-cleaning and photothermal properties owing to the intrinsic physico-chemical properties provided by graphene. This might result in high economic and environmental costs/benefits impact worldwide [119]. All of these findings evidence that protection equipment, such as face masks worn by healthcare workers and lay public, can benefit from nanomaterials, by incorporating an additional line of defense against surface- and aerosol-persistent pathogens that can result in potential infectious outbreaks [80,97]. Figure 4 shows a summary of principal coatings based on nanoparticles to prevent pathogen adhesion to surgical masks.

Another proposed strategy to improve the protective equipment and increase safety among wearers is the use of tailored nanomaterials to minimize the wettability of hydrophilic droplets by using an NP-based coating [101,120,121]. In this regard, nanofibers from different nanocomposites, such as eggshell membrane and silk fibroin nanofibers, have been evaluated, by taking advantage of their hydrophobicity and biocompatibility, to reduce the wettability in biomedical applications, such as face masks [111]. Electrospinning has been used for nanofiber fabrication and modulate the nanostructure properties, namely larger surface area, small interstitial spaces, porous structure and fiber-connectivity. It was found that hydrophilicity properties of eggshell and silk nanofibers can be tuned by changing the ratio between both nanofibers in nanocomposites [111,122]. Modifications on the nanocomposite ratio resulted in changes in the water contact angle, which is associated with the wettability of the nanofibers [111]. Similarly, aluminum doped zinc oxide (AZO) NPs have been evaluated to impart water-repellency properties when coated on polyester fabric surfaces [110]. This work showed that AZO-coated polyester nanocomposites exhibited hydrophobicity due to the surface roughness imparted by the AZO NPs resulting in a higher water repellency with a water contact angle of 146°. Furthermore, the AZO-coated polyester composites also showed UV protection capability due to the UV blocking effect provided by the ZnO NPs [110]. These results highlight the increasing need for the design of multifunctional masks to prevent the spread of infectious pathogens, such as SARS-CoV-2. The water-repellency property of the NPs shown here can be exploited in developing face masks with high hydrophobicity to prevent the persistence of virus droplets on protective equipment and therefore reduce virus transmission in care facilities as well as in public places.

### 4.3. Surface Decontamination

In addition to recommended PPE for caregivers and patients, hand hygiene and surface decontamination are also key to health safety, as the SARS-CoV-2 is known to remain viable on surfaces for hours to days [61,80,123,124]. Disinfection practices should be focused on meticulous hygiene in workspaces to minimize contaminated surfaces. As mentioned previously, nosocomial spread has been documented for infectious pathogens, including coronaviruses [59,60,73]. The multifunctionality of nanomaterials endow them the feasibility of being coated on a large number of common surfaces to act as biocidal agents for different types of pathogens, including viruses [65,66,69,102,103,104,106,107,109].

#### 4.3.1. Silver Nanoparticles

In this regard, silver nanoparticles (AgNPs) have been used as an antiseptic and disinfectant as they are able to interact with the disulfide bonds of the glycoprotein/protein contents of human pathogens, such as viruses, bacteria, and fungi, and induce their cell lysis [109,125,126,127,128,129,130,131,132]. Both AgNPs and silver ions can alter the three-dimensional structure of proteins by interfering with S-S bonds and block functional pathways of the target pathogen [133]. In the case of the virucidal activity for cell-free and cell-associated viruses, AgNPs have been reported to have size- and concentration-dependent effects on the disruption of glycoproteins of the viral envelope and act as a viral entry inhibitor [65,66,109]. The effectiveness of AgNP-based products to inactivate enveloped and non-enveloped viruses has been extended to applications to contaminated surfaces [65,66,128]. To this end, Park et al. reported the effectiveness of AgNPs complexed with magnetic hybrid colloid to inactivate three types of viruses, bacteriophage ΦX174, murine norovirus, and adenovirus serotype 2, by varying surface-water conditions after 1 h treatment [130]. Likewise, in a study assessing the effect of 3-hydroxybutyrate-co-3-hydroxyvalerate (PHBV)/AgNP fiber mats on two noroviruses, it was found that AgNPs provided a virucidal activity due to the release of silver ions from the immobilized AgNPs after 24 h exposure [128]. These findings confirm the broad spectrum of AgNPs as biocidal agents, which could also be used in coating applications to reduce the SARS-CoV-2 transmission and spread.

#### 4.3.2. Copper Oxide Nanoparticles

In addition to being cost-effective and easy to synthesize, copper oxide (CuO) NPs exhibit interesting biological properties [134]. For these reasons, they have been incorporated successfully for microorganism and virus inactivation purposes on contaminated surfaces. Thus, CuO NPs will play an important role to promote the risk reduction of people exposed to COVID-19 virus spread. In this context, Porgador et al. has studied the effect of the surfaces coated with nanoparticles of various metals on the infection rate of lentiviruses in human cells, which belong to the HIV family. Their findings show that surfaces coated with CuO NPs block the infection of the human cells by the virus. These experiments suggest that copper ions will offer huge possibilities preventing SARS-CoV-2 surface-mediated infection [135,136]. Thus, hybrid composites based on polymer/CuO NPs for antiviral coatings can be painted or sprayed on COVID-19 infected surfaces. The CuO NPs enable the controlled release of metal ions onto the coated surface. Furthermore, the release of Cu ions is slow and the coating is expected to last over weeks to months by reducing the infectivity of the virus by more than ten-fold. In a similar approach, Nanoveu, which is an Australian Singaporean-based technology company that focuses on the commercialization of ultra-functional mobile screen protectors and case technology, recently introduced the antiviral screen protector based on copper oxide nanoparticles (CuO NPs) as an antiviral material that offers a non-invasive way of protecting a user from viruses. This CuO NP functionalized surface was able to kill 99.99% of the tested viruses within minutes. This nanomaterial generates much more ROS than metallic copper NPs; these ROS are responsible for the rupture of cell walls and protein structures of viruses. This is the main mechanism through which CuO NPs damage the virus’ protective coating causing its inactivation. The coating was successfully tested through the Japanese Industrial Standard (JIS) Z 2801/ISO 22196 standards and is being currently tested on several human coronaviruses that come from the same family as SARS-CoV-2 [137].

#### 4.3.3. Titanium Oxide Nanoparticles

Titanium dioxide (TiO_2_) has also been studied owing to its biocidal properties to reduce contamination (e.g., biofouling) [138] on various types of surfaces and media [104,107,139,140,141,142,143]. Although many studies have focused on its disinfectant effects on bacteria, other studies have addressed the virus inactivation by TiO_2_, particularly at the nanoscale [104,107,144]. Specifically, the high photocatalytic property of TiO_2_ NPs has been explored to inactivate enveloped and non-enveloped viruses on different surfaces and media [104,107,139,144,145]. A case study of this was reported by Nakano et al. who described the microbicidal activity stemming from the photocatalysis of TiO_2_ NPs cast on glass surfaces against both feline calicivirus and human H1N1, a surrogate for non-enveloped virus and a surrogate for enveloped virus, respectively [104,107]. In these studies, the TiO_2_-coated glasses were manufactured using a spin-coating process with TiO_2_ followed by the International Organization of Standardization (ISO) quality testing control methodology [104,107]. The virus strains were incubated using a model mammalian cell line and classified according to their TCID50, prior to the spread of their virus titers on the TiO_2_-coated surfaces [104,107]. After illuminating the as-modified surfaces with ultraviolet A (UV-A) irradiation under 0.1 mW cm^−2^ light intensity, the results indicated that the viruses were inactivated in a time-dependent manner with the inactivation observed as early as 4 h after illumination for the human influenza virus. Similarly, the inactivation for the feline calicivirus was observed 8 h after UV exposure [104,107]. The H1N1 envelope consists of phospholipid bilayer membrane with glycoprotein spikes surrounding the capsid. A similar “corona structure” has been reported for SARS-CoV-2 [93]. This can be targeted by a photocatalytic reaction to denature the virus envelope and, subsequently, degrade the nucleic acid by the as-generated ROS [104,107]. The virus inactivation via the generation of ROS, such as·^•^OH, under UV irradiation, as the responsible mechanism for the initial protein denaturation followed by the damage at the DNA/RNA level, has also been previously reported in other TiO_2_-coated surface studies [143,144,145,146,147]. Moreover, TiO_2_ NPs can impart a self-cleaning property to the coated surfaces, thus increasing the high antiviral activity in various applications including healthcare industries [142,143,145,148]. The results summarized from these studies show that the stability of TiO_2_ under varying environmental conditions has led to its incorporation in thin films and surfaces as a biocidal agent [104,107,144,145,149]. Moreover, these studies suggest that virus titers of SARS-CoV-2 on contaminated surfaces could be quickly reduced via photocatalysis mediated by TiO_2_ NPs, due to the “corona” nature of the virus [93].

#### 4.3.4. Other Oxide Nanoparticles

Other types of oxide NPs have also been proposed for biocidal applications [104,106]. For instance, silica NPs were investigated for their potential use as an antimicrobial surface coating [106]. Hybrids between silica NPs and didodecyldimethylammonium (DDAB), a quaternary ammonium cationic surfactant, were used to coat flat surfaces to trigger a biocidal activity against bacteria (*Escherichia coli* and *Staphylococcus aureus*), yeast (*Candida albicans*), and virus (*Influenza* A/PR/8/34 H1N1), respectively [106]. After covering glass surfaces with silica NP suspensions, the results showed that silica NP-coated surfaces had a rapid and broad antibacterial, antifungal, and antiviral activity. The mode of inactivation in the case of H1N1 lied in blocking the surface adhesion of the virus titers followed by structural damage and inactivation induced by the silica NP-DDAB hybrids due to their hydrophobic composition. Notably, the biocidal properties of the silica NP-coated surfaces were not due to the leaching DDAB from the surface of the NPs but rather from the coated surfaces themselves as no DDAB residues were detected in the cell culture medium. Furthermore, the study demonstrated that the coated-silica NP surfaces can be reused with no impact on the biocidal activity over time [106]. This work showed the versatility and multifunctionality of oxide nanoparticles against various categories of pathogens while demonstrating the feasibility of reuse and sustained biocidal activity over time.

#### 4.3.5. Graphene

Associated with different materials, graphene sheets have been investigated for coating applications, such as in medical devices [150], due to their mechanical and biocidal properties [102,103,105,151]. Specifically, graphene oxide (GO) and reduced graphene oxide (rGO) sheets have been used to coat surfaces and films to deactivate pathogens including human viruses [102,103,105]. In a study looking into the antiviral properties of graphene, graphene-tungsten oxide composite thin films (0.9 nm thickness) were prepared through the chemical exfoliation method, followed by the incorporation in tungsten oxide thin film using glass substrates [105]. The films were then tested against the bacteriophage MS2 to investigate the photoinactivation potential of the films under visible light irradiation at room temperature. This resulted in the photodegradation of the viral proteins on the surface of the graphene-tungsten oxide thin films as confirmed by the inactivation of more than 99.99% of the viruses under visible light irradiation for 3 h. This inactivation was due to the breakage of the protein capsid of the viruses followed by the RNA efflux [105]. The antiviral effect of GO has been confirmed on other viruses, such as herpes simplex virus type 1, feline coronavirus and infectious bursal disease virus, which have been deactivated by sulfonated magnetic NPs functionalized with rGO [102] and GO-AgNPs [103], respectively. These results demonstrate that graphene can be used in nanocomposites or alone to impart antiviral characteristics for surface coating applications.

**Figure 5:**
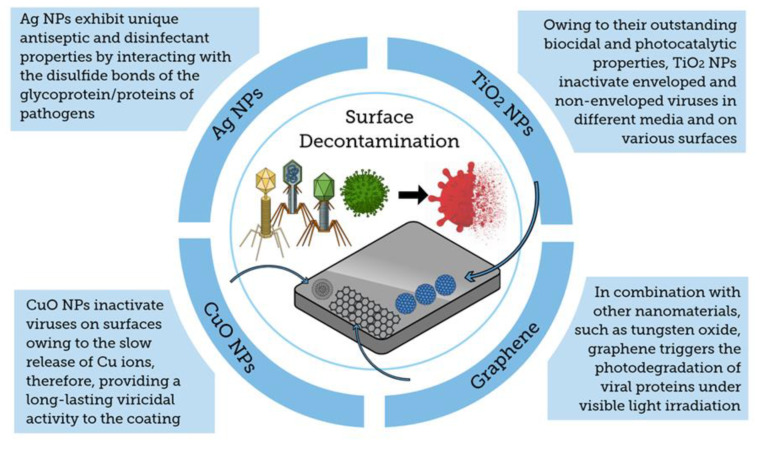
Principal coatings based on nanotechnology to prevent surface contamination by viruses and other pathogens.

Figure 5 summarizes the different types of nanoparticles used to prevent surface contamination by viruses and other pathogens.

## 5. Diagnostics and Biosensors for COVID-19

It is widely accepted that a crucial factor to impede the spreading out of the current COVID-19 or any viral diseases lies in the design of rapid, specific, and sensitive diagnostic tools since the quarantine policy has resulted in some challenges due to the lack of compliance. Therefore, there is an urgent need to provide reliable and cost-effective diagnostic tests. However, optimizing both specificity and sensitivity of these tools remains quite a challenge to overcome the risks of false positive and false negative results [152,153,154,155,156,157,158]. A second challenge to consider resides in the detection environment as the virus could be solid or liquid, in air or adsorbed on surfaces; this affects its survival characteristics. Typically, human sample specimens are collected from fluid in the upper or lower respiratory tract, feces, blood, or serum. However, there is also the need to detect the virus in the air and water due to its survival capabilities in these media that might serve as the virus vector [159,160,161]. A third challenge is associated with the need to diagnose both symptomatic and asymptomatic patients [162]. In the present section, we review the different diagnosing COVID-19 approaches, materials, methodologies, detection mechanisms, and devices used up to date.

We focus on the opportunities offered by biomedical sciences for early virus detection and diagnosis. We introduce the plasmonic phenomena which have gained considerable attention due to the outstanding sensitivity they offer in biotechnological applications, in general, and in direct immunoassay and inhibition immunoassay, in particular. We include a discussion of the Localized Surface Plasmon Resonance (LSPR) phenomena and their implementation through Surface Enhanced Spectroscopies for molecular detection. We also define the notion of chiral induced spin selectivity as a novel phenomenon based on spintronic for molecular detection. Finally, we discuss other possibilities reported in the literature, which can be potentially explored for the current and future outbreaks.

Reliable testing is crucial to both confirm current COVID-19 infected individuals but also identify individuals with infection-induced immune response. At present, there are two types of diagnostic tools that provide rapid (a few minutes) vs. slow (a few hours) results. Both have advantages and limitations due to the detection method followed. Typically, the rapid diagnostic tools are based on serological samples; on the other hand, the slow diagnostic tools are based on viral RNA samples. The viral RNA sample sensing is suitable for symptomatic and asymptomatic patients but the results may become altered when the immune system starts to react about a week after the infection. The serological sample sensing is suitable only for symptomatic patients with the immune system starting to react about 10 days after infection. The FDA has authorized the use of in vitro diagnostic products and not yet approved tests for the detection of SARS-CoV-2 antibodies for diagnosis, treatment and prevention purposes [163]. The most effective tests to support the screening, diagnosis and monitoring of COVID-19 are based on molecular serological testing of either anti-SARS-CoV-2 developed antibodies during the symptomatic phase or nucleic acids thanks to the availability of the SARS-CoV-2 genome sequence [164].

Although the term “nanotechnology” was introduced in the early 1960s, the first trials based on NPs for virus detection came out only two decades ago [165]. Designing tailored biosensors has shown significant relevance and many examples are currently successfully used in biotechnological applications [7,166,167]. Efficient biotechnological approaches relying on the use of nanomaterials for the detection and monitoring of emerging or re-emerging viral agents, such as West Nile virus, Hantavirus, Nipah virus, Chikungunya, Zika, a variety of influenza strains, Severe Acute Respiratory Syndrome (SARS) coronavirus, or Middle East Respiratory Syndrome coronavirus (MERS-CoV) [7,163,166,167,168,169] remain a key goal of current research [170]. Most of these approaches count on metallic and magnetic NPs, functional thin layers, organic/inorganic NPs, DNA nanostructures, polymer/silica NPs, quantum dots (QDs) and carbon QDs (CQDs) of different shapes, sizes and functionalities [163,171,172,173].

### 5.1. Non-Serological Methods

The most common type of molecular testing for COVID-19 uses polymerase chain reaction (PCR) from sputum or throat/nasal swabs samples. Typically, this diagnostic tool is slow and based on the detection of the genetic material of the virus. The PCR amplifies and detects DNA and RNA sequences; so far, it has become the most reliable technique in COVID-19 assessment. At present, different PCR methods have been tested to monitor COVID-19. Among the main advantages the PCR offers, one can cite time-saving, sensitivity, and minimal templates. However, most of the PCR testing requires approximately 20–40 cycles to generate enough information that enables sample analysis. Moreover, the RNA is single-stranded and unstable and presents drawbacks working with it, hence, the need to reverse transcribe it into its highly stable, complementary DNA (cDNA) using reverse transcriptase. Although the reverse transcription PCR (RT-PCR) uses the RNA as the template, it relies on cDNA for the analysis, which constitutes its main advantage. The RT-PCR also serves as the first step in quantitative PCR (qPCR) and RT-qPCR by quantifying RNA transcripts in the samples; the latter being commonly used to detect, characterize and quantify nucleic acids. Typically, RNA transcripts are quantified by, first, reverse transcribing them into cDNA, then, the qPCR is subsequently carried out. One of the qPCR advantages lies in enabling the fluorescent labeling to collect the data as the PCR progresses. The main disadvantages of these PCR-based techniques are linked to their complexity and problems associated with their sensitivity, reproducibility, and specificity. Besides, they also suffer from the main drawbacks inherent to traditional PCR when it is used as a quantitative method [163]. Bearing this in mind, it is evident that it is necessary to explore other innovative diagnostics methodologies, not only based on molecular and serological testing, but also other approaches that can play a key-role in mitigating the current COVID-19 pandemic and future virus outbreaks for testing in non-medical environments, such as airports, schools, shopping malls, etc. To date, several publications have detailed molecular methods and medical tools to accelerate the detection process of the coronavirus responsible of COVID-19 pandemic [3,7,174]. For instance, Chu et al. developed a single-step real-time reverse-transcription polymerase chain reaction (RT-qPCR) assay to detect two different regions of the viral genome [175] while González-González et al. [176] found no significant differences when they compared the efficiency of a mini-PCR equipment to its lab-bench counterpart. On the other hand, the same group designed a 3D-printed incubator for a faster amplification of the COVID-19 genome using loop-mediated isothermal amplification (LAMP) reaction that is detected using qRT-PCR [177].

COVID-19 detection can be implemented via several methodologies [3,163,178]. For instance, Zhang et al. described the use of magnetic NPs (MNPs) to extract quickly pure COVID-19 RNA for faster RT-PCR analyses [3]; these MNPs are first silanized, then coated with a negatively charged copolymer made of 1,4-butanediol diacrylate and 6-amino caproic acid monomers to give rise to highly stable, negatively charged paramagnetic pcMNPs, as confirmed by zeta-potential measurements. As a result, these pcMNPs bind more than 90% of the RNA. Moreover, the cell lysis and the RNA binding by pcMNPs are carried out within the single and same step; no elution is needed to perform the RT-PCR and the obtained results are similar to the positive control. Most importantly, this very sensitive pcMNPs-based RT-PCR method, as it displays a detection limit of 10 copies of the pseudovirus tested, is prone to automation, a key step towards high throughput viral RNA extraction and detection.

Chiral induced spin selectivity (CISS) approach could also be used to diagnose COVID-19. The CISS effect is a new, not completely understood phenomenon, allowing the creation of biomolecular and molecular scale analogs of spintronic devices. The CISS effect, firstly reported by Naaman in 1999, is based on the ability of chiral molecules to spin-polarize electrons passing through [179,180]. It is important to stress that CISS possesses variable strength depending on molecular chirality. At present, there is an increasing interest for developing more sensitive and faster detection technologies benefiting from this phenomenon due to its efficacy in detecting chiral molecules. Conversely, it is well known that the coronavirus possesses surface spike (S) proteins made of a variety of chiral oligopeptide chains that exhibit a strong CISS effect [181,182,183,184].

### 5.2. Serological Methods

#### 5.2.1. Lateral Flow Methods

Lateral flow technology has undergone tremendous developments since its first commercialization in 1984 as a urine-based pregnancy test, and has been exploited in several fields and industries for the detection of very diverse analytes, including respiratory viruses [10,185,186,187]. Owing to the presence of specific antibodies in blood samples of infected patients [188], several teams have designed lateral flow assays to rapidly and accurately detect COVID-19 without the resort to sophisticated equipment and highly skilled professionals. Typically, this rapid diagnostic is based on the molecular recognition of antibodies present in the blood or serum. However, it seems these newly developed tests have been used for validation and approval as most of the studies insist on comparing their results with RT-PCR, a routine and approved molecular method for the diagnosis of COVID-19.

A conventional molecular biology method used for the diagnosis of COVID-19 is the enzyme-linked immunosorbent assay (ELISA) which enables the detection and quantification of various water-soluble substances, such as antigens and antibodies. Li et al. used ELISA diagnosis kits based on the SARS-CoV-2 recombinant nucleoplasmid protein (rN) and spike protein (rS) by determining the presence of SARS-CoV-2 Immunoglobulin M (IgM) and Immunoglobulin G (IgG) antibodies in COVID-19 patients using horseradish peroxidase (HRP)-conjugated anti-human IgM antibody and HRP-conjugated anti-human IgG antibody, respectively [189]. As an outcome, the sensitivity of the rS-based ELISA is slightly better than the one of its rN-based analog. Moreover, the results in both cases depend similarly on days post-disease onset. Importantly, 16–20 days post-disease onset seems to be the optimal time for blood analysis posing a serious inconvenience for this method as the early stage detection of COVID-19 is critical for curing the disease and stopping its propagation. To reduce the volume of the samples analyzed, Tan et al. combined microfluidics and ELISA to detect IgG against different SARS-CoV-2 proteins in 10 µL serological samples within 15–20 min. However, the age of the used samples was not mentioned [190,191].

Using chemiluminescence immunoassay kits bearing two SARS-CoV-2 antigens that are tethered to magnetic beads, Jin et al. found out that using IgG antibodies to diagnose COVID-19 was more sensitive and specific when compared to IgM [192]. While the virus load decreased over time, IgG positive rate increased to plateau at a 100% and remained higher than the IgM all the time; on the other hand, IgM increased for a certain period before receding. This may suggest it is more accurate to rely on IgG antibodies to detect SARS-CoV-2. Although some studies confirm this trend, combining IgG and IgM signals gives even better signals (vide infra), especially that the shedding routes are multiple [178]. It is also possible to detect SARS-CoV-2 antibodies present in serum samples from people infected with other respiratory pathogens using a peptide that is attached to magnetic beads to act as the immunosorbent in a chemiluminescence enzyme immunoassay [193] with a similar magnitude of sensitivity (82.52%). In a similar way, Ashtari et al. reported the use of hybrid magnetic NPs coated with amino-modified silica (ASMNPs) fabricated by water-in-oil microemulsion method for the improved recovery of target single-stranded DNA (ssDNA) macromolecules [194]. This process provides an effective, selective and very fast method to recover specific ssDNA allowing, therefore, a highly precise sensing of diseases or mutations. Besides, the former mechanism is based on magnetic bioseparation technology which also offers unique advantages over other common, complicated and expensive traditional bio-separation technologies. The main advantage of this approach consists in offering a viable strategy that can be implemented in laboratories owing to its easy sample handling, limited reagent consumption and simple equipment necessary while it offers a better cost/benefits with a substantial economic impact. Hung et al. described an integrated microfluidic system based on functionalized 100 nm diameter manganese ferrite magnetic NPs (MnFe_2_O_4_) to perform the influenza virus detection via an immunoassay [195]. These NPs were functionalized through the layer-by-layer (LBL) surface modification to reduce the background noise. The authors also claimed that such technology can be applied in rapidly diagnosing infectious diseases. On the other hand, He et al. reported a methodology for the polymerase chain reaction (PCR) amplification based on 30 nm avidin-modified iron oxide γ-Fe_2_O_3_ magnetic NPs [196]. This alternative method can dislodge the non-target gene, therefore reducing the false positive tests, and improving the specificity of the PCR amplification. Following a similar approach, Mu et al. reported an innovative particle-based immunoassay using pentabody conjugated magnetic NPs for the rapid and sensitive detection of avian influenza virus (AIV) [197]. They combined this technology with AuNPs labelled with the anti-AIV mouse monoclonal antibody 3C8 to act as the detector. These conjugates yielded a very sensitive influenza virus detection assay that can be adapted on cheap diagnostic tests for infectious diseases. The magnetic NP morphology and shape are crucial for their performance. As an example, a gene-probe detection methodology based on magnetic/metal hybrid core-shell NP bioconjugates have been reported by Xi et al. [198]. This technology combines both the advantage of AuNPs for binding and detecting specific structures/molecules and the MNP features for the easy separation and detection. The fabrication of this core-shell NP conjugates is based on the citrate reduction of cationic gold while iron oxide NPs act as seeds creating, hence, core-shell frameworks without altering the magnetic properties of the core thanks to the metallic shell. Torres-Chavola et al. reviewed different innovative aptasensors based on NPs and aptamers which are, by definition, nucleic acid sequences that bind specific non-nucleic acid targets with a high affinity, for the detection of microorganisms and viruses [199]. The NP-based aptasensor technology does not require sophisticated devices; interestingly, the detection processes can be carried out in solution, avoiding expensive and complex pre-processing protocols. For the virus detection, RNA aptamers are very sensitive to specific hemagglutinin (HA) membrane glycoproteins which translate very well for the genome structure of other coronaviruses; therefore, this can be considered as a promising alternative to substitute complex antibodies as the recognition agents.

**Figure 6:**
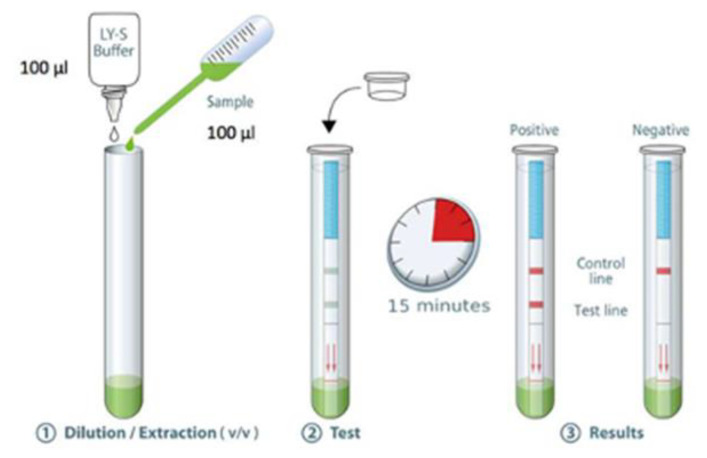
Working principle of SARS-COV-2 Respi-Strip©. Reproduced from Reference [201], under the Creative Commons Attribution License.

Xiang et al. compared the SARS-CoV-2 detection using ELISA and colloidal gold-immunochromatographic assay (GICA) kits [200]. Although both methods displayed a 100% specificity, combined ELISA IgM and IgG detection sensitivity was slightly higher when compared to GICA assay: 87.3% vs. 82.4%, respectively. Interestingly, the sensitivity values of these assays were significantly higher than the ones obtained via qRT-PCR. On the other hand, Mertens et al. developed the COVID-19 Au Respi-Strip© consisting of a nitrocellulose membrane onto which were cast AuNPs bioconjugated with monoclonal antibodies against SARS-CoV-2 [201]. Once this strip is immersed in a sample-containing test tube, the response is obtained within 15 min with a 74.2% sensitivity, a 100.0% specificity, and a 98.0% robustness, as illustrated in Figure 6.

#### 5.2.2. Surface Plasmon Resonance Techniques

**Figure 7:**
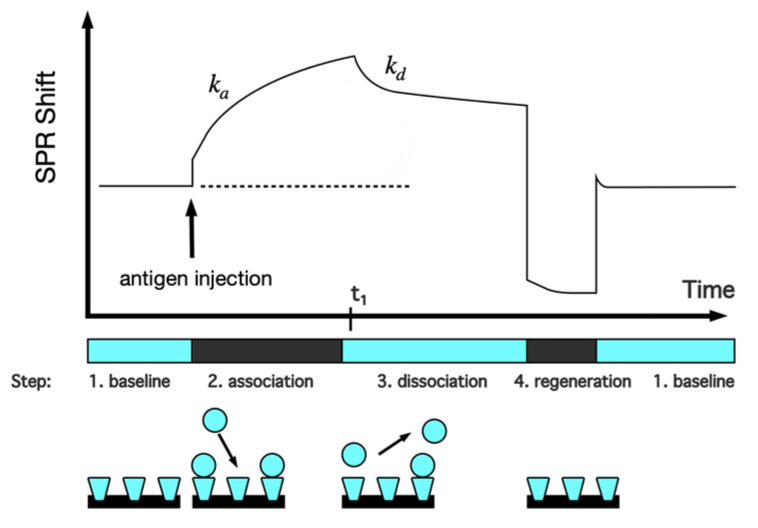
A generic cycle (sensogram) for an antigen assay including: (1) the baseline signal with no antigens present; (2) the association stage where antigens bind to the antibody; (3) the dissociation stage where some antigens are dislodged with a constant rate; and (4) the regeneration stage where the antigens are removed by spiking with pH generating the new baseline. Adapted from Ref. [210] with written permission of Dr. Richard B.M. Schasfoort.

Surface Plasmon Resonance (SPR) techniques constitute other diagnostic tools that sensitively detect refraction index changes of a surface. Currently, these label-free detection methods are considered among the best standard techniques in virology. The characterization of the binding event is presently used in basic and applied research to understand the viral infection mechanisms; this might be exploited in the development of effective diagnostic tools, such as those intended for COVID-19 [202,203]. The SPR market is increasing and has played key-roles for major companies, such as BiOptix Inc., Bio-Rad Laboratories, Nicoya Lifesciences Inc., AMETEK Inc., General Electric Co, among others. These SPR label-free or indirect detection techniques have been adopted to manufacture improved, cost-effective and versatile products in the SPR COVID-19 biosensing market for clinical applications. In terms of kinetic information, the SPR technology offers almost unique features, when compared with other available methodologies, in obtaining highly accurate results while it usually requires fewer biological reagents. Moreover, this technique that does not require significant sample preparation or sample volume can be readily automated providing fast time-to-result in addition to being particularly suitable for measuring antibodies and analytes that do not have commercially available assays. All these advantages seem to be very useful when non reliable commercial assays are available while the development of novel ELISA or immunoassays could be time-consuming [202,203,204,205]. On the other hand, the main drawback of this technique is related with the nonspecific binding which often reduces the assay sensitivity although it is of paramount importance when working with complex biological samples, like serum. However, the last can be minimized using similar approaches as those used in other commercial clinical immunoassays, such as the addition of soap to reduce undesired interactions at the surface or by coating the sample with bovine serum albumin in order to prevent sample proteins from binding. Very recently, PathSensors, Inc. announced the development of the Cellular Analysis and Notification of Antigen Risks and Yields (CANARY) biosensor to detect the novel SARS coronavirus based on SPR-exalted technologies [206,207]. The principle of SPR detection is that attached material to a thin metallic surface alters the frequency of the plasmon resonance excitation (a charge density wave) of that surface; this is detected by optical means. This change in resonance frequency can detect attached molecules onto the surface as long as these have sufficient mass or are in sufficient amounts [208]. This principle is particularly useful in the context of photothermal biosensing of COVID-19 or the antibody-antigen coupling which typically has two broad immune-assay formats: (i) direct assay where antibodies interacting with a specific antigen are attached to the metal surface yielding a particular resonance frequency. When the corresponding antigen or conjugated antigen (competition assay) attaches to the antibody, the resonance shifts and, thus, the presence of the antigen is revealed; and (ii) inhibition assay where the antigen is bound to the metal surface and the antibody is in solution. If antigens are encountered in the sample, antibodies are prevented (inhibited) from attaching to the antigens at the surface and the signal is lower than the one of the antigens originally attached. Sandwich assays mix the two previous broad types [163,204,205,209]. These assays are integrated into lab-on-a-chip approach since a complete analysis cycle of detection and regeneration can be performed [210]. Figure 7 depicts a complete cycle (a sensogram) for an antigen assay: (1) the baseline describes antibodies in the absence of antigens; (2) the association stage where the sample antigen is identified by the antibody. Here, the reaction rate k_a_ can be assessed and the antigen-antibody affinity can be determined; (3) the dissociation stage in the absence of further antigen. This stage calculates k_d_, the dissociation rate, and also determines any non-specific changes in the plasmon resonance signal; and, finally, (4) the regeneration stage where the antigens are removed from the antibodies by spiking with pH to generate the new baseline.

Beside antigen detection in the sample, time dependence is monitored to determine the association and dissociation kinetics of the bimolecular interactions. Similar to well defined capacitor, the charging and uncharging (loading/unloading) of the antigen-antibody binding determine the reaction rates k_a_ and k_d_ and the equilibrium constant K_D_ of the [A] + [B] → [AB] and [AB] → [A] + [B] reactions. This monitoring allows to deduce key features, such as the quality of the paratope/epitope match, non-specific attachment to the surface and could be coupled to a study of antibody design/optimization [163,205,208,209,211].

As widely discussed in the literature, the SPR techniques are not restricted to planar multilayers [210,212]. On the contrary, metallic NPs with dimensions much smaller than the wavelength of the exciting light can ensure a more prominent signal due to their large extinction coefficient [210], the latter being heavily dependent on the dielectric constant, size and geometry of the NPs. Thus, metallic NPs can be considered as one of the most used surface plasmon-assisted field amplifiers. Whether in suspensions or on a surface, the sensitivity of the SPR technique can be enhanced. Nevertheless, the bandwidth of the resonances is narrowed/compressed in NPs versus the continuous spectrum from zero to the ultraviolet in bulk metals. Alkali, gold and silver are by far the most used materials for SPR purposes. Besides, these metals exhibit resonances in the visible spectrum. In addition to other metals that can be useful in changing the dielectric constant of the embedding medium and developing distinct plasmon peaks [213], there exist different materials to use under different conditions [163,210,214].

Other mechanisms widely accepted to readily diagnose viruses are based on colorimetric phenomena among which the LSPR assays constitute tools of choice. The LSPR originates from the fact that noble metal NPs show a strong UV–Vis extinction band owing to the collective oscillations of their surface free electrons that are induced by an external electromagnetic wave. This field is highly localized and decays rapidly away from the NP/dielectric interface. In fact, it is well known that, in plasmonic nanomaterials, the electromagnetic field is not uniformly distributed but spatially localized in narrow regions, either (i) at the particle corners or (ii) at the interparticular gap regions. These points are usually denoted in the literature as “hotspots” which are currently extensively investigated aiming at designing highly performant plasmonic nanostructures. While growth techniques yield individual NPs that suffer from polydispersity and different geometries affecting their optimal performance, lithographic fabrication techniques offer, on the other hand, a tight control over the size and shape and the concomitant optical properties of these NPs [170,215,216].

At the wavelength of maximum extinction, the frequency is red-shifted by an increase in the dielectric constant and thickness of the material surrounding the NPs [217]. The biosensor will then work in a similar way as described before. When the antigen is bound to the surface ligands of the metallic NPs, a change in the dielectric constant of the environment is induced yielding a shift of the resonance peak of the unbound centers. As a consequence, the kinetics and equilibrium constants can be determined as previously described (Figure 7).

In this regard, Kim et al. recently reported colorimetric trials to identify targeted biomolecules through LSPR shifts and color changes in spherical AuNP suspensions by selecting a target and a control upstream of the E protein gene (upE) and open reading frames (ORF) 1a of MERS-CoV and tobacco mosaic virus (TMV), respectively [218]. This is based on the interaction of the negatively charged citrate ion-capped AuNPs with disulfides, salts and targeted probes. Interestingly, a very low limit-of-detection (LOD) was reported which makes their design of paramount importance in clinical analysis. Moreover, this methodology offered a dramatic reduction of up to 35% in the number of PCR cycles necessary to diagnose the coronavirus. In a similar approach, Li et al. demonstrated that single- and double-stranded oligonucleotides had different optical responses when adsorbed on AuNPs in colloidal solution [219]. They exploited a simple color change mechanism based on aggregation-induced electrostatic interactions to differentiate specific molecules in a colorimetric assay. Regarding the fabrication of virus detection kits, Draz et al. and Lin et al. discussed in detail the use of plasmonic AuNPs technologies to fabricate versatile virus detection tests [165,171]. These materials offer huge range, time and sensitivity improvements. Additionally, NPs have also served in the recognition of biomolecules, such as DNA, aptamers, and antibodies, or in the fabrication of complex bioconjugates, such as: (i) antibody–AuNPs, (ii) DNA–AuNPs, (iii) artificial ligand–AuNPs, and (iv) enzyme–AuNPs to detect and monitor the coronavirus [165,171]. A few years ago, Huang et al. reported the detection of nucleocapsid proteins of SARS-coronavirus based on 20 nm diameter AuNP-bioconjugates [220].

**Figure 8:**
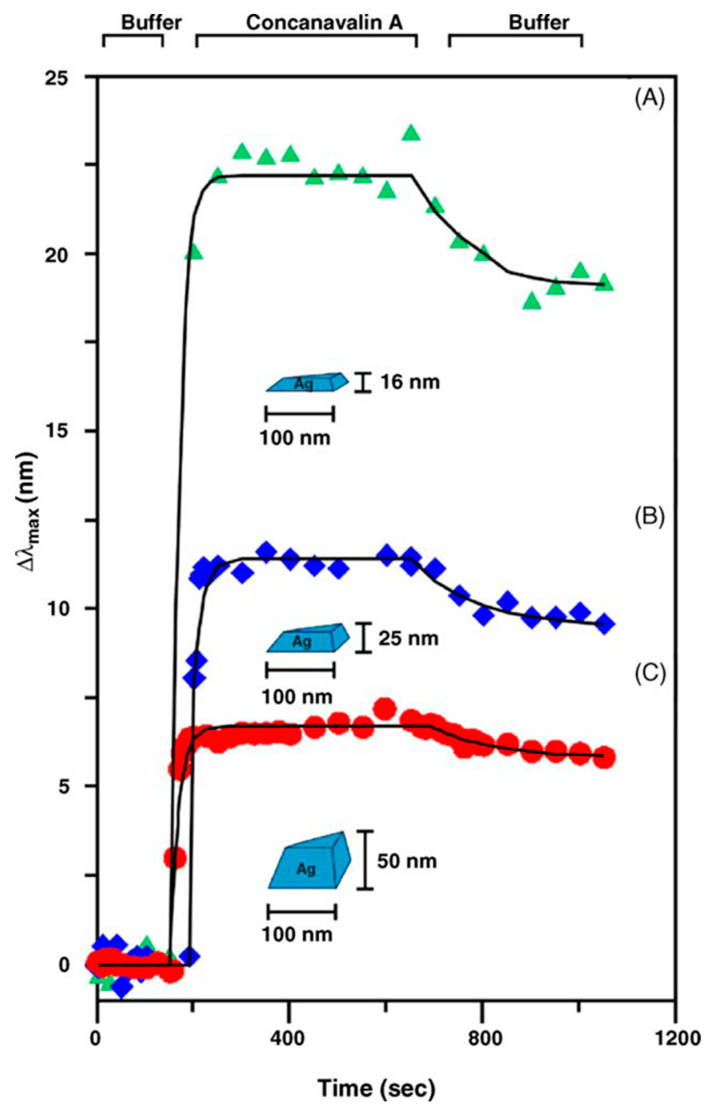
Real-time response (cycle) of Ag-conjugated nanosensor for a typical carbohydrate-binding protein at (**A**) 16 nm out-of plane height, (**B**) 25 nm out-of plane height, and (**C**) 50 nm out-of plane height from. Reproduced from Reference [216] with permission from Elsevier.

In a similar way, Yonson et al. reported the fabrication of AgNP-based LSPR nanosensors for carbohydrate-binding protein [216]. Figure 8 describes the real-time (cycle) response of this Ag-conjugated nanosensor showing the same stages than a direct assay explained in Figure 7 for AgNP triangular patterns of different heights. Furthermore, the dependence of the LSPR response on particle morphology enables the optimization of the sensors by controlling the NP size and shape.

#### 5.2.3. Raman Spectroscopy Techniques

Another approach to study biological systems is based on Raman Spectroscopy, one of the most valuable vibrational spectroscopies in identifying specific signatures of small molecules in a given sample. Nevertheless, due to the small cross section of small molecules, high intensity lasers or prolonged data acquisition are required. One way to overcome such problems is by combining Raman spectroscopy with LSPR giving rise to Surface Enhanced Raman Spectroscopy (SERS), a subset of Raman spectroscopy. Large local electromagnetic fields generated by LSPR produce dipoles in molecules present on patterned metal surfaces and enhance cross sections of the target molecules [221]. SERS offers up to a million-fold enhancement by means of plasmonic nanostructures providing unequaled detection sensitivity up to the single molecule level. SERS effect is achieved via two distinct mechanisms, that is, (i) electromagnetic enhancement (EM) and (ii) chemical enhancement (CHEM). The EM often provides the strongest contribution with an enhancement factor typically in the range of 10 to 100 compared to CHEM [189,222]. To mention some of its most common applications in biological systems, SERS offers the advantage to be used in two ways either as (i) direct (label-free) detection or (ii) indirect detection. The former is very easy to implement but often yields poor sensitivity due to the intrinsic properties of the analyte. The latter needs tailored SERS tags/probes which provide a response from the target bound-ligand or from the environmental properties; it also sacrifices partially the molecular information but allows qualitative and quantitative sensing due to the stable signals coming out from the SERS probes. Thus, SERS provides enhanced efficacy reducing the need for powerful lasers and crucial information raising from the environment surrounding the specimen through live-kinetics SERS characterization. In this context, SERS-based immunoassays with high sensitivity up to the picogram in microfluidic setups have been achieved [223]. Relying on antigen assays, antigen-antibody interactions have been studied to determine the blood fungal pathogens [224]. Moreover, multiple viral antigen detection with SERS active nanoparticles at sensitivities of fg mL^−1^ has been reported [163,205]. Very recently, Zhang et al. reported the use of SERS for the ultra-fast and onsite detection of COVID-19 in environmental samples [167].

#### 5.2.4. Colorimetric Techniques

**Figure 9:**
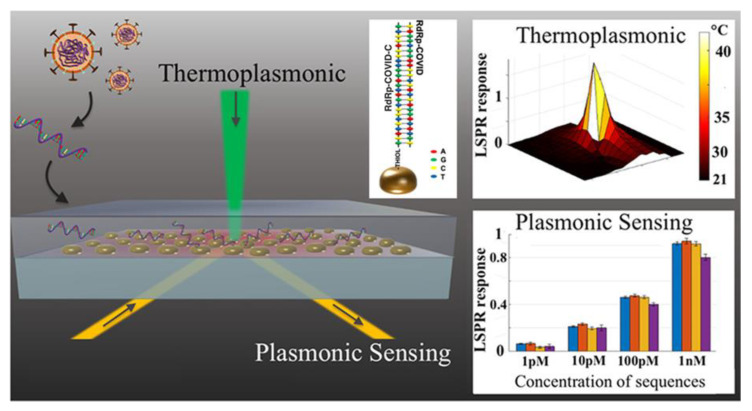
Working principle of SARS-CoV-2 sensor based on two-dimensional (2D)-surface Au nanoislands conjugated with thiolated cDNA acting as the capture probe: once DNA hybridization takes place owing to the thermoplasmonic effect, the induced Localized Surface Plasmon Resonance (LSPR) response is correlated to the concentration of the targeted sequences of SARS-CoV-2. Adapted from Reference [212] with permission from the American Chemical Society (ACS).

An electrochemical biosensor measuring the current as a function of the bound amount of SARS-CoV-2 antigen has been reported [225]. If the genome amplification is required, this might be easily achieved by coupling the loop-mediated isothermal amplification (LAMP) method to AuNP-based colorimetric biosensor for COVID-19 diagnosis [226]. Adding up to this knowledge, Li et al. developed a point-of-care lateral flow immunoassay (LFIA) that detects simultaneously the presence in human blood of IgG and IgM due to COVID-19 within 15 min owing to the interaction of these antibodies with SARS-CoV-2 recombinant proteins—acting as antigens—that are adsorbed onto the surface of 40 nm-colloidal AuNPs [189]. The sensitivity of this assay increases tremendously when IgM and IgG screening is carried out at the same time. This SPR mediated immunoassay showed improvement of the sensitivity, i.e., positive cases over clinical positive samples, as it reaches up to 89%; concomitantly, the specificity was also improved, i.e., false positive cases over clinical negatives cases, as it reaches up to 90%. Notably, its detection consistency is 100% as this assay gives the same results for COVID-19patients and healthy persons regardless of the blood sample types (fingerstick blood, serum, plasma of venous blood). A similar test, Viva-DiagTM was designed by Paradiso et al. to screen the health workers of their Cancer Institute (Bari, Italy) and determined that the proportion of COVID-19 infected patients among an asymptomatic cohort was very small (1.1%) [227]. However, their results seem to be non-conclusive as a chemiluminescence LFIA gave very different results. Additionally, Chen et al. devised another LFIA test using lanthanide-doped NPs as a signaling agent to detect SARS-CoV-2 IgG in human serum [228]. This assay proved to be rapid (10 min), sensitive, specific and reproducible. Furthermore, SARS-CoV2 was detected using sensor chips based on gold nanoislands (AuNIs) labeled with thiol-cDNA that acts as a capture probe when the complementary DNA is added [212] (Figure 9). The hybridization process takes advantage from the photothermal effect made possible by the excitation of these AuNIs by a 32-mW laser at 532 nm wavelength applied in the normal incident angle avoiding, therefore, the use of an external source of heat. On the other hand, the second sensing laser (at the same 532 nm wavelength) is placed at an incident angle of 72°. Concomitantly to the hybridization, the LSPR spectrum is recorded. This dual-functional plasmonic photothermal bioplatform enables both a fast hybridization kinetics and a very sensitive detection of exclusively SARS-CoV-2 complementary DNA sequence achieving a detection limit of 0.22 pM.

To enhance the fluorescence signal, Shen et al. reported a sensitive signal amplification based on LSPR hierarchical optical antenna effect consisting of an inverse opal (IO) crystal modified with AuNPs [229]; this gives rise to a 3D photonic scaffold which allows both light flow control and light harvesting. This nanostructure transforms the incident light into a standing wave promoting intense localized fields on the AuNPs yielding improved light-use efficiency. This setup revealed to be suitable for label-free DNA detection of SARS offering, additionally, signal and detection intensity enhancement. Martinez-Paredes et al. also reported a SARS coronavirus genosensor designed on a disposable gold nanostructured screen-printed carbon electrode [230].

#### 5.2.5. Nanoparticle-Based Biosensing

Other noble metals have been used successfully for virus diagnosis and detection. Specifically, AgNPs are among the most employed plasmonic nanostructures for biomedical applications [135,231,232,233,234]. The main advantage of AgNPs lies in their higher extinction coefficient when compared to AuNPs. This promotes signal enhancement besides its concomitant optical sensitivity for colorimetric assays. In this context, Teengam et al. reported the fabrication of a coupled colorimetric and paper-based analytical devices (PADs) for DNA detection based on AgNP aggregation induced by acpcPNA (pyrrolidinyl peptide nucleic acid) [233]. This nanodevice was tailored to detect synthetic oligonucleotide targets from MERS-CoV sequence. This technology is particularly attractive for virus sensing due to its readily applicable methodology and lack of sophisticated scientific equipment; this constitutes a huge advantage especially in low-income countries where no adequate equipment nor skilled personnel are available in healthcare facilities. This technology would be of particular interest for the current COVID-19 and futures outbreak as it offers the possibility to fabricate simple, affordable, portable and disposable diagnosis tests. Another interesting approach can be inspired from the work of Li et al. where they detailed a versatile H1N1 influenza virus detection protocol based on 40-nm AgNP conjugates [235]. This sensor, based on the chemiluminescent (CL) metallo-immunoassay method, enables the detection of molecules at a very low concentration via a mechanism that exhibits a high performance without any sophisticated signal amplification. This is based on a combined enzyme-linked immunosorbent assay (ELISA) and luminol-conjugated AgNPs. The authors of this study claim their methodology is much more sensitive than others, such as the electrochemical ones, or the conventional Inductively Coupled Plasma Mass Spectrometry (ICP-MS). It is worth mentioning that not only the type of metallic NP employed is a key parameter for the detection assay improvement but also their morphology. In this regard, a very interesting hybrid approach for the multiplexed DNA detection was proposed by Sha et al. using molecular beacons adsorbed on nanowires made of alternating gold/silver segments [236]. This technology is very useful in pathogen detection assays as it combines hybrid metal-bioconjugated nanomaterials and the fluorescence quenching ability offered by the metallic surfaces. This strategy achieves a high selectivity in the detection of pathogens, such as SARS, from a multiplexed RT-PCR without any sophisticated instrumentation beyond a fluorescence microscope.

**Figure 10:**
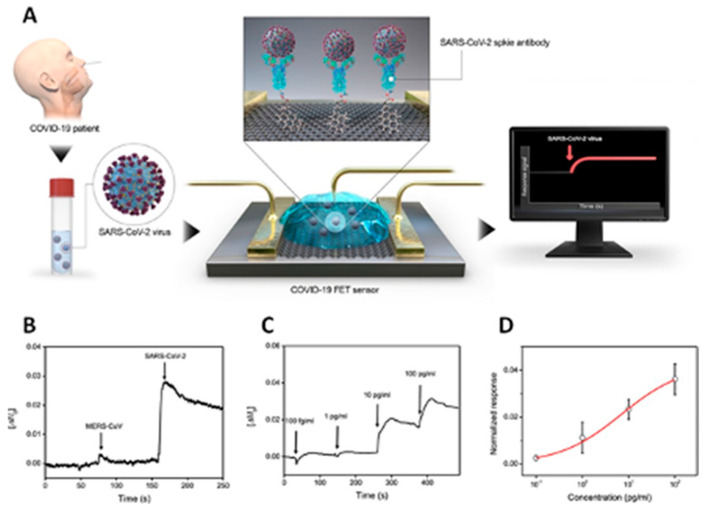
COVID-19 field-effect transistor biosensor: (**A**) working principle; (**B**) specificity to SARS-CoV-2; (**C**) current response as a function of antigen concentration; and (**D**) correlation curve between current response and antigen concentration. Adapted from Reference [238] with permission from the American Chemical Society.

Beside metallic NPs, other organic, inorganic or natural nanostructures have also been screened for the fabrication of biosensors for the early virus detection. For instance, Ishikawa et al. reported the fabrication of an indium oxide (In_2_O_3_) nanowire-conjugate based biosensor where the capture agent is an antibody mimic protein (AMP), a special kind of polypeptides that bind to their target with high affinity and specificity in a very similar way to regular antibodies [237]. AMPs are relatively small structures; besides, they exhibit a very good stability in a wide range of pH and electrolyte concentrations making them particularly attractive for biosensing applications. In this work, the authors relied on this technology using fibronectin (Fn) as the AMPs to detect the nucleocapsid (N) protein—a well-known SARS coronavirus biomarker—at subnanomolar concentrations. Additionally, other nanomaterials, such as carbon-based nanostructures, have been reported for sensing applications related to COVID-19. For instance, Seo et al. demonstrated the fabrication of an innovative biosensing device based on the field-effect transistor (FET) technology for SARS-CoV-2 detection [238]. They used a nanocarbon FET with a 100 m^2^ effective working area where the detection mechanism is based on the coupled antibody-conjugated graphene. Graphene layers were fabricated via conventional wet-transfer methods and monitored the selective real-time FET response to differentiate between the targeted SARS-CoV-2 antigen protein and MERS-CoV proteins as its electrical response can be exclusively correlated to the amount of SARS-CoV-2 antigen, as shown in Figure 10. The sensor performance was assessed using antigen protein, cultured virus and nasopharyngeal swab specimens from clinical samples. This approach offers a simple and rapid sensing platform providing a highly responsive detection of the SARS-CoV-2.

Multiplexed diagnosis mechanism based on dendrimer-like DNA (DL-DNA) nanostructures for DNA detection was reported by Li et al. [239]. In this technology, DL-DNA structures were used as the fluorescent dye to construct fluorescence nanobarcodes. To this aim, a typical Y-DNA structure composed of a sticky end consisting of three oligonucleotides (complementary to each other) and a fluorophore or a molecular probe on the other two ends was fabricated. This enabled the univocally detection of small DNA fragments of SARS coronavirus using fluorescence microscopy, dot blotting, and flow cytometry. This strategy offers many advantages; the DL-DNA can be used as both a structural scaffold and a probe while its detection mechanism relies on fluorescent color ratios instead of single color and can be easily adapted to a fluorescence detection system. Moreover, Roh et al. reported a testing protocol using the system on a chip (SoC) technology whose detection mechanism is based on QDs-conjugated RNA aptamer [240]. The amine group of the RNA aptamer was covalently attached to the commercially available, fluorescent carboxyl terminated QDs605, usually made of a nanometer-scale CdSe core and ZnS shell to better control and modulate their optical response. Such a system enables the immobilization and detection of the SARS-coronavirus nucleocapsid (N) protein on the surface of a glass chip. This innovative approach might be easily adapted to the current SARS-CoV-2 pandemic. Other bio-hybrid nanomaterials might also be of interest to tackle COVID-19, such as the ones based on silica that have been used successfully for biotechnological applications including bioimaging and biosensing, and pathogen detection [173,241]. It is expected that more approaches for the diagnosis of COVID-19 will be reported in the coming weeks and months yielding a tremendous boom in this field as it always happened after recent outbreaks [242,243,244,245,246].

## 6. Treatments for COVID-19

Thus far, more than 1700 clinical trials related to COVID-19 have been registered worldwide, the USA leading with more than 300 trials among which a substantial majority is sponsored by the National Institute of Allergy and Infectious Diseases, followed by France, Spain, Italy, Germany, China, UK, Canada, and Switzerland. The majority of the clinical trials deal with anti-malarial drugs including hydroxychloroquine which represent more than 20% of the trials. Among the drugs tested, some slow the infection progression while others mitigate the risk of complications. Currently, there is no approved therapy by international regulatory bodies for the treatment of COVID-19. Different treatments in tests are summarized in the WHO database. Among the current recommendations, the WHO advises that investigational drugs, approved or authorized agents for other indications, can be accessed and prescribed through various mechanisms, including the U.S. Emergency Use Authorizations.

### 6.1. Computational Modeling for COVID-Related Structures

**Figure 11:**
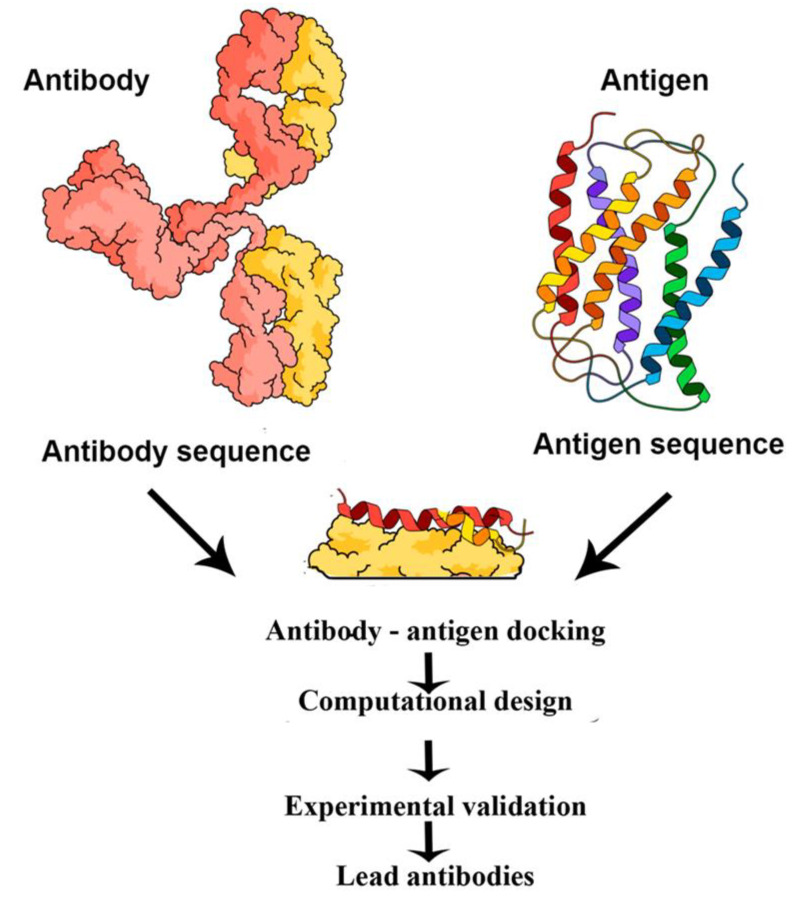
Block diagram for computational modeling in antibody-antigen docking.

Computational methods are widely regarded as useful tools in understanding and predicting small molecule active agents in drug development for an efficient targeting and tuned specific effects. The screened drugs and molecules through optimized computational methods might be very useful in fighting COVID-19, as reported recently [247,248]. These results can help in guiding empirical testing during the drug discovery process. When it comes to aiding antibody designs for antigen specificity, one is confronted with the need to simulate complex proteins made up of convoluted peptide segments. To address these structures as a whole, the computer power is just not available for classical simulation/molecular dynamics and even less so for ab-initio approaches. It is then inevitable to take the same route as for the drug discovery process by identifying possible key regions of the protein that are critical in antigen identification. The hypervariable region amino acid sequence of the heavy-light variable domain of the antibody is a relatively short sequence that can be used for simulation and modeling [249]. Figure 11 illustrates the antibody-antigen docking as well as the typical block diagram in computational modeling. It is worth mentioning that antibody designs must deal with the complementary-determining region (CDR) whose sequence is highly variable due to the genetic process that yields the specific chain segment at the surface of the B Cells. The CDRs are short sequences of amino acids that are found in the hypervariable domains of proteins with the function of antigen receptor that complements the antigen and gives that receptor its specificity.

In a typical computational modeling, the possible zones of interaction between the variable regions of the antibody and the amino acids that make up the epitope of the antigen are considered. The CDRs from the antibody come into contact with the antigen and the particular amino acid sequence with its bends and winds either make a tight fit to the antigen residues—areas of the antigen denominated epitopes—and bind through weak colloidal forces or not [250]. The description of the hypervariable region and the potential antigen sites to form paratope-epitope pairing are the targets for computational simulation [251]. The amino acid sequence and the corresponding structure to complement the antigen complex is the main challenge for the computational approach [252]. In the absence of known antigen-antibody interactions, the conjectures will come from the immune response of patients who have recovered, and the computational approach attempts to improve on the existing antibodies. Moreover, the simulations will access different antigenic target structures on the viral surface.

The complexity of the biological structures involved in the relatively short sequences of the hyper-variable sequence of amino acids, whose role is to meet an exponential variety of antigen challenges, is mostly addressed by Molecular Dynamics (MD) [252,253]. Very recently, Pant et al. described in-silico approaches to identify possible protease inhibitors against SARS-CoV-2, using MD simulations on four selected compounds from the CHEMBL database to validate the stability of their interaction with the main protease of SARS-CoV-2 (Mpro). They also performed MD simulations on the protein data bank (PDB) structure 6YF2F to understand the differences between screened molecules and co-crystallized ligand [247]. On the other hand, Kumar et al. reported the successful identification through MD simulations of phytochemical inhibitors against the main protease of SARS-CoV-2. Namely, natural metabolites, such as ursolic acid, carvacrol and oleanolic acid, are potential inhibitors of SARS-CoV-2 main protease (Mpro). According to their findings, these molecules have passed the 4 pharmacokinetic steps (Absorption, Distribution, Metabolism, and Excretion) as well as the Lipinski rule of five. These results coming from MD simulations suggest that the three phytochemicals studied could serve as potential inhibitors of SARS-CoV-2 main protease and control the viral replication [248]. It is important to mention that, to extract reliable predictions from MD simulations, one must consider both sampling configurations produced in the course of time and accurate classical force fields that are used in the equations of motion of the amino acid chains. If the sampling of configurations is not sufficient, the results will not be very useful.

Sampling: some very ingenious methods have been devised for improved sampling protocols, namely the Replica Exchange Molecular Dynamics (REMD) method that uses replicas of the same system at different temperatures in order to assay the potential energy space more efficiently. Alternating with lower temperature replicas, one can analyze local minima with more subtlety [254].

Force Fields: on the other hand, high precision force fields have been made available with the Assisted Model Building with Energy Refinement (AMBER) [255], Optimized Potentials for Liquid Simulations (OPLS) [223] and Chemistry at Harvard Macromolecular Mechanics (CHARMM) [256]. Other open source force fields are available through GROningen MAchine for Chemical Simulations (GROMACS) [257] and Large-scale Atomic/Molecular Massively Parallel Simulator LAMMPS [258].

It is worth mentioning that there are plenty of online web servers, such as Web Antibody Modeling (WAM) [259] and Prediction of Immunoglobulin Structure (PIGS) [260], which enable computational modeling of antibody variable regions. Likewise, Rosetta Antibody is a novel antibody variable region (FV) structure prediction that incorporates sophisticated techniques to minimize the complementarity determining regions (CDRs) loops and optimize the relative orientation of the light and heavy chains, as well other models that predict successful docking of antibodies with their unique antigen [261]. Another strategy gaining interest deals with artificial intelligence (AI) to obtain the design of COVID-19 antibodies [262] and would be an adequate route to find appropriate highly stable complementary antigen-antibody paired sites [263].

### 6.2. Artificial Intelligence

Artificial intelligence (AI) allows to analyze the immense amount of information generated every second to predict the behavior of the infection in the world by monitoring millions of variables related to the coronavirus from mathematical models, projections, new outbreaks, the behavior of industry-specific shoppers, web searches for people with COVID-19 symptoms, worldwide clinical trials and real-time news [264]. The Canadian AI company, BlueDot, has several software products capable of predicting abnormal outbreaks of diseases with infectious potential of massive infections throughout the world. This company was able to alert their clients of an abnormal increase in pneumonia cases in Wuhan 9 days before the WHO warned the world about COVID-19 [264].

On the other hand, the Massachusetts Institute of Technology assures that AI can be used to determine the spread of the pandemic indirectly, as simple as analyzing the data of the purchases made by citizens of one country or another on Amazon. Items related to face masks and PPE peaked in sales, first in Italy, then Spain, France, United States, and Canada. Furthermore, the level of spread can be inferred by analyzing the most used keywords in relation to COVID-19 symptoms in search engines and the number of downloads of healthcare applications in different locations. The reported cases of coronavirus were also compared with airline ticket sales to predict the spread of the virus and take quarantine measures [265].

Big data management allows the real time monitoring of the outbreak. The information available today in combination with real-time information in the location of people and mathematical models are the perfect combination to generate predictions that help protecting the most vulnerable and making data-driven political decisions [266]. Yang and colleagues showed that a 5-day delay in applying restrictive measures was the key to unleashing the global pandemic. Furthermore, their mathematical models warn of new outbreaks after the relaxation of isolation and social distancing measures [267].

Other researchers have been able to apply pattern recognition to computed tomography (CT) lung scans and automatically diagnose the presence or absence of COVID-19 infection. The investigations verified the effectiveness of the software diagnosis by corroborating the presence or not of the virus with PCR tests [268]. InferVision, a Chinese startup, applies this technology in 45 hospitals from China, the USA and Europe and has performed more than 76,000 tests [269].

AI technology can also hasten the discovery of SARS-CoV-2 treatments and vaccines. For example, Benevolent AI, from the UK, searches, among the existing drugs, for the interaction they would have with the new coronavirus and their effectiveness through AI. Benevolent AI has unraveled the antiviral properties of Baricitinib, a rheumatoid arthritis drug, which is now in phase III clinical trials for COVID-19 treatment [270]. A similar scope is being applied by researchers from the UTE University (Quito, Ecuador) who have analyzed in silico 3885 immune system proteins to reveal potential therapeutic targets for drug repurposing finding 75 potential therapeutic targets with promising results. Besides, Insilico Medicine, a Chinese startup, uses AI to discover new drugs to combat COVID-19. This company creates “deepfakes” for molecules by applying the RGA technology, a new technology used for “deepfakes” (manipulated media content completely realistic) or to create photographs of people who do not exist as a sufficient database of faces can enable the AI to create new faces. As a result, Insilico Medicine has identified more than 100 new molecules, with capabilities to inhibit the coronavirus among which six are being manufactured to evaluate their clinical efficacy [270].

**Figure 12:**
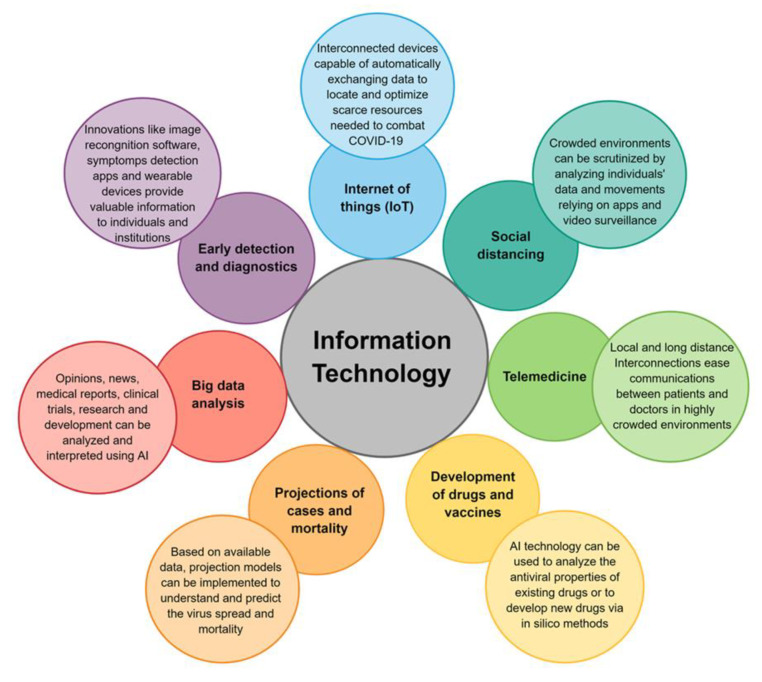
Main applications of information technology in combating COVID-19.

Megvii, another Chinese startup specialized in facial recognition, has implemented an algorithm that is capable of detecting patterns in people with suspected symptoms of infection in crowded places, such as high fever or cough by analyzing facial features and temperature. Experts affirm that in the long term this technology will be useful in the development of smart cities with the help of AI and internet of things (IoT). This could prevent the spread of new viruses or pandemic pathogens that hit society in the same way that COVID-19 has done, increasing the traceability monitoring and prevention measures [264,271]. A summary of the principal tools from Information Technology (IT) is presented in Figure 12.

### 6.3. Small Molecule Therapeutics

In the current context, researchers are working against the clock to combat COVID-19. According to a recent article, more than 160 medications at different stages of development are being investigated to fight the coronavirus [14]. The reuse of existing drugs is thought to be a more time-efficient and cost-effective strategy compared to discovering new drugs and vaccines [17]. Anti-malarial drugs are among the most popular molecules including hydroxychloroquine, hydroxychloroquine sulfate, chloroquine, chloroquine phosphate, ivermectin, to name a few. The second category of popular drugs are antiviral molecules as they inhibit the replication of the virus genome; it includes Remdesivir, Ritonavir, Lopinavir, Favipiravir, Oseltamivir, among others. The candidate antiviral agent Remdesivir (Gilead Labs, Foster City, CA, USA), designed to deal with the Ebola outbreak in 2013, and tested with viruses that did not end in a pandemic, is being used for the treatment of COVID-19 in hospitalized patients [272]. The third category of popular drugs are antineoplastic or immunomodulatory agents to adjust the immune response caused by COVID-19 that encompasses, for instance, Baricitinib, Tocilizumab, Sarilumab, Ruxolitinib, and Anakinra. These therapeutics can specifically target the virus as this works with the use of serum from individuals who have suffered from the disease and have a good antibody response [273]. Alternatively, modulators of the immune response, such as interleukin 6 (IL6) inhibitors [273] or interferons, have enabled promising results [274]. The last category of drugs consists of antibiotics, such as Azithromycin, to modulate the inflammatory response. While single drug treatments seem to have some positive results with some molecules, it is clear that drug combinations (double or triple) hold more promises due to the need to both inhibit the spread of COVID-19 in the body and improve the patient’s health by mitigating other complications, such as the severe inflammation that damages the organs. Among the possible promising drug combinations, the use of hydroxychloroquine sulfate and chloroquine phosphate together with Remdesivir was approved for certain hospitalized patients with severe COVID-19 when a clinical trial is unavailable or is not feasible [18]. Although many existing treatments for other related conditions are being tested and some combinations have been successfully used for specific populations, no universal therapies have been achieved for COVID-19.

### 6.4. Main Research Lines in Anti-COVID-19 Vaccines

**Figure 13:**
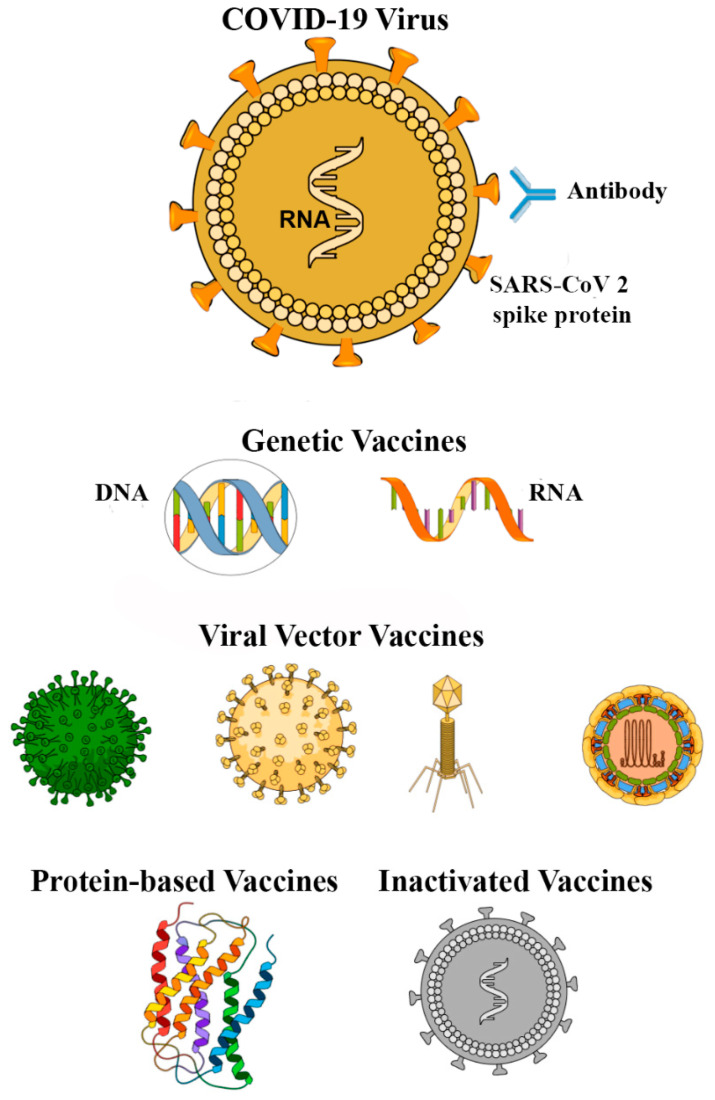
Different design strategies for new vaccine candidates based on nucleic acids such as DNA or RNA, viral vectors of different origins, peptide sequences or the antigen of the inactivated COVID virus.

The surface glycoproteins of the SARS-CoV-2 are the primary target for vaccine development. One of the primary goals of human COVID-19 vaccines is to elicit a strong humoral immune response against the S protein [275]. Moreover, MERS-CoV-specific CD4+ and CD8+ T cells are detected in peripheral blood mononuclear cells (PBMC) in MERS-CoV-infected survivors [276]. This suggests that a balance between B and T cell responses is essential in the treatment of MERS-CoV and SARS-CoV-2 infections. In this sense, different strategies are being developed to obtain a lasting response of B and T cells for SARS-CoV-2 and MERS-CoV. Among these are traditional live attenuated, inactivated, and subunit vaccines, and the latest development of nanoparticles conjugated to other antigens, vector vaccines, and RNA/DNA vaccines (Figure 13) [277]. In SARS-CoV-2 infection, antibodies against the S protein were shown to be protective in multiple animal studies [278].

RNA vaccines exhibit several advantages over protein or DNA vaccines [279]. In RNA vaccines, the patient’s cells act as a factory to produce the antigen that will induce the immune response, with the necessary tertiary structure and even with the optimum degree of glycosylation, avoiding the purification of proteins in the conventional models that rely on producing recombinant proteins. Furthermore, the RNA itself acts as an adjuvant to enhance the immune response. Nevertheless, the future will tell which type of vaccine is the most effective against SARS-CoV-2.

Since the onset of the current COVID-19 pandemic, the international scientific community has been working actively on the development of vaccines. To date, there are 145 possible vaccine candidates of which 21 are in clinical trials in humans on 7 July 2020 [280].

Viral inactivation is the fastest way to develop vaccines in emergencies, such as the present one. Traditional ways of achieving this reside in inactivating the virus either by physical or chemical methods, or even by combined approaches [278]. Inactivated or weakened vaccines have been developed for diseases, such as measles, chickenpox, and polio. These successful vaccines have been tested for years. The fundamental problem with a new design lies in the non-negligible occurrence of a protective immune response.

The Chinese company Sinovac Biotech has been testing an inactivated vaccine called CoronaVac [281]. In June 2020, the company announced phase I/II trials with 743 volunteers and reported no serious adverse effects or immune response. Sinovac then launched phase III trials in Brazil in July 2020. The company is also building a facility to annually manufacture up to 100 million vaccine doses. Moreover, an agreement is reached with the United Arab Emirates to begin testing the efficacy of its inactivated virus vaccine in this country [281].

Protein subunit-based vaccines are considered the safest form among the vaccines [279]. However, the low immunogenicity of subunit vaccines dictates a high dependence on adjuvants [281]. Instead of showing the full pathogen to B cells, protein subunit vaccines only display the body parts of the virus [282]. For COVID-19, most vaccine developers look for the spike protein that SARS-CoV-2 uses to enter the human cells. The hope is that by showing the characteristic protein, B cells will also be able to recognize it in the pathogen. Protein subunits cannot become a full-blown infection but the immune response they induce weakens over time meaning people may need boosters throughout their life. Some seasonal flu vaccines take the form of protein subunits, just like the human papilloma virus (HPV) vaccine. So far, none of the protein subunit vaccines has been tested in humans [283]. For the elaboration of a protein subunit, the genetic engineering of host organisms, such as *E. coli*, to insert the genetic material coding for this protein of interest is required. Once the protein is produced and purified, the appropriate adjuvants, that make the immune system cells recognize these proteins as foreign objects and produce an immune response, must be selected [284]. To expedite this process, the intermediate step of antigen production in a host cell can be eliminated as human cells themselves are capable of doing so once the genetic material that codes for these proteins is injected.

Nucleic acid vaccines use double-stranded DNA (dsDNA) or messenger RNA (mRNA). Once the genome of a new pathogen is sequenced, scientists can isolate the target proteins for the body to recreate. However, the challenge resides in making the body responding to these target proteins [285]. Nucleic acid vaccines made from DNA have to pass through the cell membrane and the nuclear membrane; the latter protecting its DNA. Although mRNA vaccines only have to pass through the cell membrane, an additional hurdle arises: even if the cells make the desired protein, they have to fold these biomolecules in a way that resembles the actual viral protein. In this context, GeoVax is currently conducting three clinical trials in different phases using its GeoVax’s Modified Vaccinia Ankara virus-like particle (GV-MVA-VLPTM) vaccine platform and vaccine experience to design and construct vaccine candidates using genetic sequences of SARS-CoV-2. GeoVax’s Modified Vaccinia Ankara platform creates non-infectious virus-like particles in inoculated individuals. The genetic sequences of the target antigens are inserted into the MVA genome yielding their expression in infected cells. The platform also aggregates sequences that incorporate antigens into VLP and, at the same time, promote their release from the membranes of infected cells.

mRNA-1273 is a SARS-CoV-2 mRNA vaccine encoding a stabilized prefusion form of the spike (S) protein, developed by Moderna in collaboration with researchers from the Vaccine Research Center (VRC) of the US NIAID [286]. mRNA technology commonly employs nanoparticle-based drug delivery approaches. In this technology, the stretch of RNA required to prepare the vaccine is first synthesized and then embedded in lipid nanoparticles (LNPs). mRNA-1273 consists of an mRNA pharmacological substance that is manufactured in LNPs composed of the patented ionizable lipid, SM-102, and 3 commercially available lipids, cholesterol, 1,2-distearoyl-sn-glycero-3-phosphocholine (DSPC) and 1,2-dimyristoyl-rac-glycero-3-methoxypolyethyleneglycol-2000 (DMG-PEG2000). Moderna’s mRNA vaccine against COVID-19 has entered Phase III trials in July 2020, raising the hope to have vaccine doses ready by early 2021 [287].

## 7. Conclusions and Perspectives

The last decades have witnessed increasing interest and tremendous developments in biomedical science and engineering through adaptative, collaborative, multidisciplinary, and innovative research efforts made by private and public institutions to meet the challenges that constantly arise including the current, unprecedented COVID-19 pandemic; this has significantly benefited human health and society. For example, efficient and effective monitoring, detection and prevention of viral outbreaks, along with the development of treatments and vaccines, have been achieved; this great progress has enabled to save countless lives. However, there are still several technological challenges that hinder infield clinical applications, requiring further multidisciplinary investigations. The former and current SARS-CoV outbreaks highlight the importance of implementing health policies and developing technologies based on basic and applied sciences. All of the topics discussed, in detail, in the present review, are very useful for research in the field of biomedical science and engineering, to tackle the current COVID-19 pandemic and any future outbreaks, not only from an experimental point-of-view, but also from approaches relying on computational simulations, artificial intelligence, and smart devices. Nanotechnology is one of the most important fields that can significantly impact the development of protecting, diagnosis, and therapeutic solutions. The synthesis and/or discovery of nanomaterials with advanced properties and unique functionalities would elicit formidable advances in the development of such solutions. Moreover, future perspectives in biomedical science and nanomedicine applied to viral diseases are expected to mainly focus on (i) early, portable, highly sensitive, and affordable fabrication of diagnosis kits, avoiding complex infrastructures, sophisticated devices, and skilled professionals, and (ii) development of theranostic nanomedicine tools, including the development of biodegradable vectors suitable to current patients’ treatment. Along with computational simulations and artificial intelligence, a substantial contribution is expected to stem from the tandem synthetic biology and protein engineering for the development of powerful therapeutic solutions. Furthermore, reliable and cost-effective biomedical devices are also expected to provide affordable, practical, and easy-to-use products accessible for all countries worldwide, with a special emphasis on low-income countries. Finally, apps, software, internet, and smart technologies are outstanding tools to monitor, control, and predict the evolution of COVID-19 pandemics.

## Figures and Tables

**Table 1:** Contemporary innovations in the field of biomedical devices to tackle the current COVID-19 pandemic, as well as future pandemics.

Innovation	Description	Source	Type
Intensive care unit (ICU) from containers	Shipping containers repurposed to create biocontainment ICU units.	[39]	Medical facility
Stanford Pneumask	Reusable full-face snorkel mask	[40]	Personal Protection Equipment (PPE)
Maker Mask	Three-dimensional (3D) printable respirator quality masks	[41]	PPE
3D shield	3D printed powered air purifying respirator (PAPR) created at Duke University	[42]	PAPR
Massachusetts Institute of Technology (MIT) origami face shield	Single piece disposable face shields for mass production	[43]	PPE
Epidax	National University of Singapore portable on-site COVID-19 polymerase chain reaction (PCR) diagnostic system	[44]	Diagnostic test
Glassafe	Transparent material to make close proximity safer. Used in aircraft to isolate passenger seats.	[45]	Social distancing
MIT Emergency ventilator	Open-source, low-cost ventilator	[46]	Ventilator
Resuscitation bags low-cost ventilator	Resuscitation bag adaptation to build an emergency low cost ventilator (Georgia Tech)	[47]	Ventilator
Ventilator Intervention Technology Accessible Locally (US National Aeronautics and Space Administration (NASA) VITAL Ventilator)	Emergency ventilator prototype developed in 37 days	[48]	Ventilator
Harvard-MIT detection mask	Face mask that lights up a fluorescent signal in the presence of SARS-CoV-2	[49]	PPE and diagnostic

## Abbreviations

ACE-2	Angiotensin-Converting Enzyme 2
AIDS	Acquired Immunodeficiency Syndrome
AI	Artificial Intelligence
CANARY	Cellular Analysis and Notification of Antigen Risks and Yields
CDC	US Center for Disease Control and Prevention
cDNA	Complementary DNA
CDR	Complementary-Determining Region
CISS	Chiral Induced Spin Selectivity
CL	Chemiluminescence
COVID-19	Coronavirus Disease 2019
ELISA	Enzyme-Linked Immunosorbent Assay
ePPE	Electronic PPE
FDA	US Food and Drug Administration
GISAID	Global Initiative on Sharing All Influenza Data
GPS	Global Positioning System
GROMACS	Groningen Machine for Chemical Simulations
H1N1	Influenza A virus subtype H1N1
H9N2	Influenza A virus subtype H9N2
HIV	Human Immunodeficiency Virus
IgG	Immunoglobulin G
IgM	Immunoglobulin M
IoT	Internet of Things
ISO	International Organization for Standardization
LAMMPS	Large-scale Atomic/Molecular Massively Parallel Simulator
LAMP	Loop-Mediated Isothermal Amplification
LFIA	Lateral Flow Immunoassay
LOD	Limit of Detection
LSPR	Localized SPR
MD	Molecular Dynamics
MERS	Middle East Respiratory Syndrome
NIAID	National Institute of Allergy and Infectious Diseases
NMs	Nanomaterials
NPs	Nanoparticles
OSHA	US Occupational Safety and Health Administration
PCR	Polymerase Chain Reaction
PIGS	Prediction of Immunoglobulin Structure
HPV	Human papillomavirus
PPE	Personal Protective Equipment
QDs	Quantum Dots
qPCR	Quantitative PCR
ROS	Reactive Oxygen Species
RT-PCR	Reverse Transcription PCR
RT-qPCR	Reverse Transcription Quantitative PCR
SARS	Severe Acute Respiratory Syndrome
SARS-CoV-2	Severe Acute Respiratory Syndrome Coronavirus 2
SERS	Surface Enhanced Raman Spectroscopy
SIV	Swine Influenza Virus
SPR	Surface Plasmon Resonance
ssDNA	single-stranded DNA
TMV	Tobacco Mosaic Virus
VLP	Virus-Like Particle Platform
VRC	Vaccine Research Center
WAM	Web Antibody Modeling
WHO	World Health Organization
